# Nanocurcumin-Loaded UCNPs for Cancer Theranostics: Physicochemical Properties, In Vitro Toxicity, and In Vivo Imaging Studies

**DOI:** 10.3390/nano11092234

**Published:** 2021-08-29

**Authors:** Anbharasi Lakshmanan, Roman A. Akasov, Natalya V. Sholina, Polina A. Demina, Alla N. Generalova, Ajithkumar Gangadharan, Dhiraj K. Sardar, Krishna Bharat Lankamsetty, Dmitry A. Khochenkov, Evgeny V. Khaydukov, Sergey V. Gudkov, Manonmani Jayaraman, Senthilselvan Jayaraman

**Affiliations:** 1 Department of Nuclear Physics, Guindy Campus, University of Madras, Chennai 600025, Tamil Nadu, India; anbhulaksh118@gmail.com; 2 I M Sechenov First Moscow State Medical University, 119991 Moscow, Russia; sholinanv@gmail.com (N.V.S.); khaydukov@mail.ru (E.V.K.); 3 Federal Scientific Research Center, “Crystallography and Photonics”, Russian Academy of Sciences, Leninskiy Prospekt 59, 119333 Moscow, Russia; angeneralova@gmail.com; 4 Shemyakin-Ovchinnikov Institute of Bioorganic Chemistry, RAS, 117997 Moscow, Russia; polidemina1207@yandex.ru; 5 Department of Physics and Astronomy, University of Texas at San Antonio, San Antonio, TX 78249, USA; akgsh2005@gmail.com (A.G.); Dhiraj.Sardar@utsa.edu (D.K.S.); 6 Department of Natural Sciences, Texas Agriculture and Mechanical University, One University Way, San Antonio, TX 78224, USA; 7 Federal State Budgetary Scientific Institution “Federal Scientific Agroengineering Center VIM” (FSAC VIM), 109428 Moscow, Russia; krish.agudguy@gmail.com; 8 FSBI “N.N. Blokhin National Medical Research Center for Oncology”, Ministry of Health of the Russian Federation, Kashirskoe Shosse 24, 115478 Moscow, Russia; khochenkov@gmail.com; 9 Medicinal Chemistry Center, Togliatti State University, Belorusskaya Str. 14, 445020 Togliatti, Russia; 10 Biophotonics Center, Prokhorov General Physics Institute of the Russian Academy of Sciences, Vavilova St. 38, 119991 Moscow, Russia; s_makariy@rambler.ru; 11 Department of Closed Artificial Agroecosystems for Crop Production, Federal State Budgetary Scientific Institution “Federal Scientific Agroengineering Center VIM” (FSAC VIM), 5 First Institutskiy pr-d, 109428 Moscow, Russia; 12 Department of Chemistry, Quaid-E-Millath Government College for Women, Chennai 600002, Tamil Nadu, India; jmanon32@gmail.com

**Keywords:** upconversion nanoparticles, theranostics, nanocurcumin, herbal drugs, intravital imaging

## Abstract

Formulation of promising anticancer herbal drug curcumin as a nanoscale-sized curcumin (nanocurcumin) improved its delivery to cells and organisms both in vitro and in vivo. We report on coupling nanocurcumin with upconversion nanoparticles (UCNPs) using Poly (lactic-co-glycolic Acid) (PLGA) to endow visualisation in the near-infrared transparency window. Nanocurcumin was prepared by solvent-antisolvent method. NaYF_4_:Yb,Er (UCNP1) and NaYF_4_:Yb,Tm (UCNP2) nanoparticles were synthesised by reverse microemulsion method and then functionalized it with PLGA to form UCNP-PLGA nanocarrier followed up by loading with the solvent-antisolvent process synthesized herbal nanocurcumin. The UCNP samples were extensively characterised with XRD, Raman, FTIR, DSC, TGA, UV-VIS-NIR spectrophotometer, Upconversion spectrofluorometer, HRSEM, EDAX and Zeta Potential analyses. UCNP1-PLGA-nanocurcumin exhibited emission at 520, 540, 660 nm and UCNP2-PLGA-nanocurmin showed emission at 480 and 800 nm spectral bands. UCNP-PLGA-nanocurcumin incubated with rat glioblastoma cells demonstrated moderate cytotoxicity, 60–80% cell viability at 0.12–0.02 mg/mL marginally suitable for therapeutic applications. The cytotoxicity of UCNPs evaluated in tumour spheroids models confirmed UCNP-PLGA-nanocurcumin therapeutic potential. As-synthesised curcumin-loaded nanocomplexes were administered in tumour-bearing laboratory animals (Lewis lung cancer model) and showed adequate contrast to enable in vivo and ex vivo study of UCNP-PLGA-nanocurcumin bio distribution in organs, with dominant distribution in the liver and lungs. Our studies demonstrate promise of nanocurcumin-loaded upconversion nanoparticles for theranostics applications.

## 1. Introduction

Cancer is a major global health issue that needs novel approaches in addition to conventional surgical resection, chemotherapy, or radiotherapy [1]. Over the past decade, by the advancement in BioNanoMedicine, nanoparticles with various natures have attracted more attention as cancer theranostics agents that combine both visualization and therapy modalities. Drug carriers based on nanoparticles have lower side effects compared to the free drug, demonstrate increased tumour-specific targeting as a result of the enhanced permeability and retention (EPR) effect, and can provide controlled drug release, which makes the use of nanodrug delivery systems a promising approach in bionanomedicine [2].

Rare earth-doped upconversion nanoparticles (UCNPs) are a unique category of optical nanomaterials that exhibit anti-Stokes process of converting the near infrared radiation (NIR) to visible/NIR emission [3]. UCNPs demonstrate unique optical properties, including high penetration into biological tissues due to so called near-infrared transparency window, high resistance to photobleaching, absence of autofluorescence, multicolour emission with a narrow line width, high luminescence lifetime, low irradiation damage, and increased signal-to-noise ratio and photochemical stability [4]. These properties make UCNPs an excellent platform for biovisualization. However, UCNPs could additionally be loaded with antitumor drugs to obtain a theranostics platform with both visualization and therapeutic properties [5].

Curcumin is a yellow coloured polyphenol compound that has many promising biomedical properties like anti-oxidant [6], anti-fungal effect [7], anti-inflammatory, anti-proliferative [8], antiviral [9] effects, etc. It has been reported that therapeutic efficacy of curcumin is useful in the treatment of digestive and reproductive system cancers, haematological and brain tumours, urological cancer, breast cancer, and bone tumours [10]. The main problem of curcumin is hydrophobicity, which leads to poor water solubility, low absorption, quick drug metabolism, low penetration, and degradation [1,7,11,12,13]. The oral bioavailability of curcumin is limited, as it gets unabsorbed due to little intestinal absorption capability. Even the slightly absorbed part rapidly metabolized. Therefore, rapid metabolism and excretion causes certain difficulties in using curcumin for biomedical and clinical applications [11,14,15,16,17,18,19]. To solve the aforesaid problems, encapsulation of the curcumin to nanoformulations is of great interest. The efficacy of curcumin nanoformulations has been demonstrated earlier. Rupesh Kumar Basniwal et al. evaluated the anticancer properties of nanocurcumin in the presence of cancer cell lines such as lung (A549), liver (HepG2) and skin (A431) cancer cells and nanocurcumin exhibited increased anticancer properties over the conventional curcumin formulations [20]. Here, we propose nanocurcumin as model antitumor drug for loading into the nanocomplexes based on UCNPs for theranostics applications.

Although various synthesis methods were reported to prepare NaYF_4_ UCNPs, pharmaceutically important microemulsion synthesis strategy is important for drug delivery applications and therefore this synthesis methodology was adopted in the present work. It is able to provide sustained drug release, improved drug solubility and enhanced drug absorption. Specifically, the reverse microemulsion (water-in-oil, hydrophilic-lipophilic balance < 10) is an effective method to prepare hexagonal NaYF_4_:Yb,Er/Tm UCNPs [21,22,23]. Poly (lactic-co-glycolic acid) (PLGA), which is known for attractive biocompatibility, non-toxic nature and high stability, has been proposed to enhance the surface properties of UCNPs within nanocurcumin loading. Drug delivery of curcumin accompanied by PLGA could increase therapeutic efficacies to promote sustained drug delivery and drug release targeting, minimize the non-specific consumption by undesirable tissues and to enhance the aqueous solubility [10,24,25]. Adeeb Shehzad and co-workers reported so as curcumin incorporated PLGA nanoparticles have exhibited increased cellular uptake, induced the apoptosis and suppressed tumour cell proliferation and improved the bioavailability [10].

The advantage of combining UCNP and nanocurcumin is that the upconverted visible light could excite the photosensitizer and favour fluorescence resonance energy transfer (FRET). The UCNPs could also applicable for photodynamic therapy and photothermal therapy of cancers. NIR excitation based cancer therapy using UCNP that lies in the biological windows is advantageous than quantum dots and other fluorophores. The NIR activation of UCNPs could accomplish photothermal conversion and provide deep tissue penetration ability [3,26]. The rare-earth doped NaYF_4_ is attractive in biomedical applications as it gives high upconversion quantum yield than the quantum dots by two-photon energy transfer process from the sensitizer ion and the activator ion.

We report on synthesis and characterisation of biofunctional nanocomplexes of NaYF_4_:Yb,Er and NaYF_4_:Yb,Tm upconversion nanoparticles surface-coated with PLGA and nanocurcumin. The phase formation, morphology and basic upconversion emission properties of the synthesised UCNP complexes were investigated. Besides, in vitro cytotoxicity of the biofunctional nanocomplexes was tested using rat glioblastoma cells. In vivo biodistribution of these nanocomplexes was investigated in small animals.

## 2. Methodologies

### 2.1. Materials

The curcumin (C_21_H_20_O_6_) and Poly(D,L-lactide-co-glycolide) (Mw~30−60 kDa, lactide:glycolide 50:50) were purchased from Sigma-Aldrich, Saint Louis, MO, USA. Dichloromethane (CH_2_Cl_2_, SRL, Chennai, India), tetrahydrofuran (C_4_H_8_O, Alfa Aesar, Haverhill, MA, USA, 99.99% purity), ethanol (C_2_H_5_OH, 99.99% purity), sodium fluoride (NaF, Chennai, India, 99%), yttrium nitrate Y(NO_3_)_3_·6H_2_O (Alfa Aesar, Haverhill, MA, USA, 99.99%), ytterbium nitrate (Yb(NO_3_)_3_.6H_2_O, Alfa Aesar, Haverhill, MA, USA, 99.99%), erbium nitrate (Er(NO_3_)_3_·5H_2_O, Alfa Aesar, Haverhill, MA, USA, 99.99%), thulium nitrate (Tm(NO_3_)_3_·6H_2_O, Alfa Aesar, Haverhill, MA, USA, 99.99%), isooctane (SRL, Chennai, India 99.8%), oleic acid (OA, Sigma-Aldrich, Saint Louis, MO, USA, 90%), cetyltrimethylammonium bromide (CTAB) (Sigma-Aldrich, Saint Louis, MO, USA, 95%), 1-butanol (Vetec, Sigma-Aldrich, Saint Louis, MO, USA, ≥99.5%), and acetone (Rankem, Mumbai, India, 99%) were used in the synthesis of UCNPs. All chemicals and solvents are in analytical purity.

### 2.2. Preparation of Nanocurcumin

Nanocurcumin was synthesized using the evaporation-assisted solvent–antisolvent method in the Optical Nanomaterials Laboratory, Department of Nuclear Physics, University of Madras, Chennai, India. Tetrahydrofuran (THF) was chosen as solvent and distilled water act as antisolvent. The 0.1 g of curcumin was dissolved with 1:10 ratio of THF and distilled water. The sample was stirred for 20 min to acquire homogeneous solution. Subsequently, the resultant mixture was subjected to ultra-sonication (20–20,000 kHz) in the water bath-sonication for 150 min to produce an emulsion and rapidly stirred for 30 min. The supernatant was decanted and a thick residue was air-dried by 80 °C heating to about 3 h.

### 2.3. Preparation of Nanocurcumin Loaded UCNPs by PLGA Polymer

To prepare UCNP1-PLGA-nanocur composite, the hexagonal NaYF_4_: 20% Yb, 2% Er UCNPs was synthesized by reverse microemulsion method based on the earlier reports with a minor alteration in calcination temperature of 550 °C [23]. Further, the drug loading process was done based on the molecular interaction method. A weighed amount of 80 mg of NaYF_4_:Yb,Er-550 °C (UCNP1) nanoparticles and biocompatible polymer PLGA (50:50) taken with an amount of 25 mg be liquefied in dichloromethane (DCM) under mild stirring. Then, 50 mg of nanocurcumin was added with the above polymeric solution, and the yellowish solution was stirred vigorously. The concentration of PLGA is kept low to avoid the upconversion emission quenching by polymer functional groups. Then, the suspension was stirred well for 4–5 h continuously to allow the drug molecules to dissolve and be adsorbed on the UCNPs. Later, after the homogenization, the combined DCM/organic phases are evaporated. Further, the precipitated sample was obtained by washing with ethanol. The supernatant was isolated by filtration from the drug loaded nanocarrier to measure the amount of free drug. Finally, the sample was air dried overnight without any heating to avoid degradation of the polymer. The collected yellowish orange solid of nanocurcumin drug loaded samples were characterized. Similarly, to synthesis the UCNP2-PLGA-nanocur composite, NaYF_4_:Yb,Tm was prepared by doping 2% of Tm(NO_3_)_3_·6H_2_O on the contrary to 2% of Er(NO_3_)_3_·6H_2_O and calcined at 550 °C for 30 min. Then, 25 mg of biocompatible polymer PLGA (50:50) and 80 mg of nanocurcumin was added in DCM under stirring to conjugate the UCNPs. All the above upconversion nanomaterials were prepared in Optical Nanomaterials Laboratory (J.S. Lab.), Department of Nuclear Physics, University of Madras, Chennai, India,

### 2.4. Materials Characterizations

In order to analyse the crystal structural phase of the UCNPs and nanocurcumin drug loaded UCNP samples, powder X-ray diffraction (XRD) study was carried out with the BRUKER D8 ADVANCE X-ray Diffraction platform (Karlsruhe, Germany) at Cu Kα1 X-ray wavelength of 0.15406 nm. The X-ray diffraction data was collected in the two-theta range from 5 to 65° at a scanning rate 0.03 s^−1^. XRDA software (version 3.1, http://ccp14.cryst.bbk.ac.uk/ccp/ccp14/ftp-mirror/xrda/pub/lpsd/, accessed on 16 August 2021) was used for the diffraction profile fitting analysis. The surface morphology of the UCNP, UCNP-PLGA and UCNP-PLGA-Nanocurcumin was examined by High Resolution Scanning Electron Microscope (FEI Quanta FEG 200F, Hillsboro, OR, USA). Image J software (https://imagej.nih.gov/ij/download.html, accessed on 16 August 2021) was employed to calculate the particle size. Energy Dispersive X–ray spectroscopy Analysis (EDAX) and elemental mapping of NaYF4:Yb,Er-PLGA-Nanocurcumin complex was performed using the ZEISS Field Emission Scanning Electron Microscope (GeminiSEM 300, Oberkochen, Germany), which equipped with EDX detector (ULTIM MAX Silicon Drift Detector, OXFORD INSTRUMENTS, High Wycombe, United Kingdom), and the results are provided in the Supplementary Information. The thermal behaviour of the raw-curcumin and synthesized nanocurcumin was explored by Differential Thermal Analysis (DTA) and the temperature dependent weight change property was studied by Thermogravimetric analysis (TGA) by heating the sample from room temperature to 500 °C at a scanning rate 10 °C min^−1^ using the TGA-DTA Thermal Analyser (STA 2500 Regulus Simultaneous Thermal Analysis system, NETZSCH, Selb, Germany) under the nitrogen atmosphere, the results are given in Supplementary Information. The chemical functional group analyses of the samples were investigated by employing the Bruker FTIR Spectrometer ALPHA II (Ettingen, Germany) in the infrared absorption frequency range 500–4000 cm^−1^. Raman spectra of the UCNPs, UCNP-PLGA and UCNP-PLGA-Nanocurcumin samples were explored by interacting the samples with diode pumped solid state laser at 532 nm using the Horiba Jobin Yvon XPloRA Plus Raman Microscope (Horiba Techno Service, Ltd., Kyoto, Japan) in the spectroscopic mode. Optical absorption of the as-received curcumin and the solvent-antisolvent synthesized nanocurcumin was studied using the Analytik Jena UV-VIS spectrometer (Specord 210 Plus, Jena, Germany) and the spectral results are given in the Supplementary Information. Photoluminescence behaviour of curcumin and nanocurcumin was studied with HORIBA FluoroMax Plus spectrofluormeter (Techno Service Co., Ltd., Kyoto, Japan) by exciting the samples at 424 nm wavelength and the emission results are provided in the Supplementary Information. The hand-held 200 mW 980 nm infrared diode laser pointer was employed to check upconversion emission at each stage of sample preparation. For the detailed investigation of the upconversion emission characteristics of the UCNP, UCNP-PLGA and UCNP-PLGA nanocomplexes, we employed the sophisticated Quanta Master 51 spectrofluorometer (Photon Technology International Inc., Birmingham, NJ, USA) and 980 nm laser excitation source (Spectra Physics, Model 3900S, Milpitas, CA, USA). The down converting NIR fluorescence was recorded by InGaAs detector (Teledyne Judson Technologies, 062-8451, Montgomeryville, PA, USA). Zeta potential of the UCNP, UCNP-PLGA and UCNP-PLGA-Nanocurcumin was measured the HORIBA Nanoparticle Analyzer (nanoPARTICA SZ-100, Horiba, Ltd., Kyoto, Japan), and the results are available in the “Supplementary Information”. The ChemSpider free online chemical structure database (https://www.chemspider.com/StructureSearch.aspx, accessed on 16 August 2021) was employed to draw the chemical structure of THF, PLGA and curcumin.

### 2.5. Biomedical Investigation

#### 2.5.1. Cell Culture

Rat glioma C6 cells were cultivated in Dulbecco’s Modified Eagle Medium (DMEM). To make the complete medium, it was supplemented with 10% foetal bovine serum (FBS), 2 μM L-glutamine, and a combination of streptomycin (100 μg/mL) and penicillin (100 U/mL) antibiotics. Cells were grown at 37 °C in a 5% CO_2_ humidified atmosphere and passaged every 2–3 days at 80–90% confluence. Passaging was performed with 0.25% Trypsin–EDTA solution, and the subcultivation ratio was 1:3 to 1:6 according to ATCC recommendations.

#### 2.5.2. Cytotoxicity in Monolayer Culture (MTT Assay)

Rat C6 glioma cells (5 × 10^3^) were placed on a 96-well flat bottom plate and incubated for a night at 37 °C in a 5% CO_2_ humidified atmosphere. UCNPs were suspended in full DMEM just prior to the experiment and added to the cells to the final concentrations of 0.02–0.8 mg/mL for 48 h. After that, MTT (3-(4,5-dimethylthiazol-2-yl)-2,5-diphenyltetrazolium bromide) solution (0.5 mg/mL) was added to the cells for 3 h. Then, MTT solution was removed and replaced with 100 µL of DMSO. The absorbance of the dissolved formazan was measured with a microplate reader (Multiscan, Thermo FS, Massachusetts, United States of America) at a wavelength of 570 nm according to the manufacturer’s protocol. All measurements were performed in three independent replicates, and the final data were indicated as average ± SD, and the absorbance of the non-treated (control) cells was taken as 100%.

#### 2.5.3. Cytotoxicity in Tumor Spheroids (MTS Assay)

Tumour spheroids were obtained using liquid overlay technique as per the previous report [27]. For this, we resuspended 1.5% wt of agarose in PBS (pH 7.4) and heated the mixture in water bath for 15 min that resulted in agarose melting and sterilization. After that, we placed 100 μL of agarose solution to a flat-bottom 96-well plate under sterile conditions. Then, plates with agarose were cooled down to room temperature for 15 min, which led to agarose solidification and non-adhesive surface formation. We seeded rat C6 glioma cells onto obtained agarose-coated plates (10,000 cells/well in 100 μL of full DMEM) and incubated for 72 h in standard culture conditions to generate spheroids. After that, UCNPs suspensions in full DMEM were added to spheroids to the final concentration of 0.12–0.8 mg/mL for 48 h. The cytotoxicity was evaluated using colorimetric MTS assay. For this, we added 10 µL of MTS reagent per well and incubated for 3 h at 37 °C, and the soluble formazan was measured at 490 nm with a microplate reader (Multiscan, Thermo FS, Massachusetts, United States of America) according to the manufacturer’s protocol.

#### 2.5.4. Intracellular Accumulation (Anti-Stokes Photoluminescence Microscopy and Confocal Fluorescence Microscopy)

Rat glioma C6 cells (5 × 10^4^) were seeded on an 8-well glass chamber slide and incubated overnight. Then, UCNPs were suspended in full DMEM to 0.05 mg/mL and added to the cells for 1 h. After that, the cells were thoroughly rinsed three times with sterile PBS (pH 7.4) and finally fixed using 4% formaldehyde solution. For fluorescence microscopy, the cell nuclei were additionally stained with doxorubicin (100 µM, 10 min). The excitation wavelength for nanocurcumin was 488 nm, and the emission signal was collected in 500–600 nm.

#### 2.5.5. Live-Dead Assay on Tumour Spheroids (Confocal Microscopy)

Tumour spheroids were generated as it was described above. Then, the spheroids were incubated with 0.4 mg/mL NaYF_4_:Yb,Er-PLGA-nanocur and NaYF_4_:Yb, Tm-PLGA-nanocur for 48 h. Spheroids were stained with Calcein AM (50 µM) and Propidium Iodide (25 µM) for 30 min at 37 °C in a 5% CO_2_ humidified atmosphere. Stained spheroids were rinsed with PBS (pH 7.4), mounted on a glass slide, and analysed using Leica TSP SPE confocal microscope.

#### 2.5.6. Accumulation in Tumour Spheroids (Confocal Microscopy)

Tumour spheroids were generated as it was described above. Then, the spheroids were incubated with 0.4 mg/mL NaYF_4_:Yb,Er-PLGA-nanocur and NaYF_4_:Yb,Tm-PLGA-nanocur for 2 h and 48 h. Then, spheroids were washed with PBS (pH 7.4), mounted on a glass slide, and analysed using Leica TSP SPE confocal microscope. The excitation wavelength for nanocurcumin was 488 nm, and the emission signal was collected in 500–600 nm.

#### 2.5.7. Animal Experiments

The experimental animals (female BDF1 mice) were kept under controlled environmental conditions, in particular constant temperature, humidity, and a 12 h dark–light cycle. They were allowed free access to water and mouse chow. All animal experiments were performed in accordance with European and Russian national guidelines for animal experimentation, and animal experiments were approved by the local animal and ethics review committee of the FSBSI “N.N. Blokhin Russian Cancer Research Center”.

#### 2.5.8. Biodistribution In Vivo

The experiments were carried out on female BDF1 mice (C57Bl/6 × DBA2). Before the UCNPs administration, mice were pre-anesthetized with a combination Zoletil 100 (2.5 mg/kg tiletamine hydrochloride and 2.5 mg/kg zolazepam hydrochloride) (Virbac, Caro, France) and Rometar (xylazine hydrochloride 0.2 mg/kg) (SPOFA, Markova, Czech Republic) intraperitoneally, 150 μL/mouse. The drug was administered intravenously through the retro-orbital sinus at a dose of 0.75 mg/mouse (30 mg/kg). Mice were sacrificed by instantaneous dislocation of the cervical vertebrae in the cranio-cervical direction 4 h and 24 h after intravenous administration. Post-mortem parenchymal organs were exterminated and images were obtained.

#### 2.5.9. Lewis Lung Cancer Mouse Model

Lewis lung cancer (LLC) model BDF1 (C57Bl/6 × DBA2) mice was proposed to evaluate the UCNPs accumulation in tumours in vivo. For this, LLC was extracted from BDF1 mice (day 11 after grafting) and subcutaneously inoculated into experimental mice (0.3 mL LLC in DMEM suspension 1:10). After two weeks, the UCNP suspension (0.75 mg in 150 µL) was injected into the mice peritumorally. Images were obtained from the DVS-02 imaging system 24 h after administration.

## 3. Results and Discussion

### 3.1. Nanocurcumin-Loaded UCNPs Preparation

**Figure 1:**
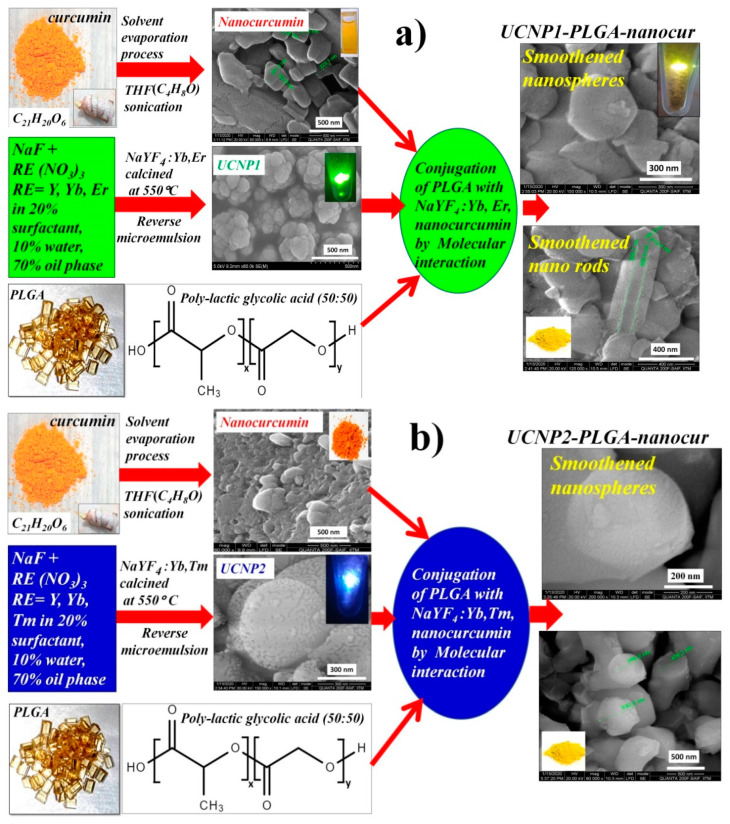
Formation mechanism of (**a**) UCNP1-PLGA-nanocur, (**b**) UCNP2-PLGA- nanocur.

Figure 1a,b depicts the schematic synthesis of UCNP1-PLGA-nanocurcumin and UCNP2-PLGA-nanocurmin. The loading of nanocurcumin into UCNP involved three stages. Firstly, nanocurcumin was prepared by using the solvent-evaporation assisted solvent-antisolvent method using THF and distilled water. It showed absorbance at 425 nm and emission at 560 nm (Figure S1a,b; detailed discussion is given in the supplementary information). The sample was collected after solvent evaporation by sonication and air-dried at 80 °C to remove water moiety, which is confirmed through the TGA and DTA results shown in Figure S2a,b. In the second step, NaYF_4_:Yb,Er (UCNP1) and NaYF_4_:Yb,Tm (UCNP2) nanoparticles were synthesized utilizing the reverse micro-emulsion process with strategy of surfactant/water/oil phase solvents. It yielded spherical nanospheres suitable to load the nanocurcumin. Finally, UCNP1-PLGA-nanocur and UCNP2-PLGA-nanocur composites were prepared by facile molecular interaction approach. The PLGA was used to encapsulate the nanocurcumin into UCNPs to improve the surface properties by interacting with the carboxylic groups of the polymer [20,28].

### 3.2. HRSEM Analysis

**Figure 2:**
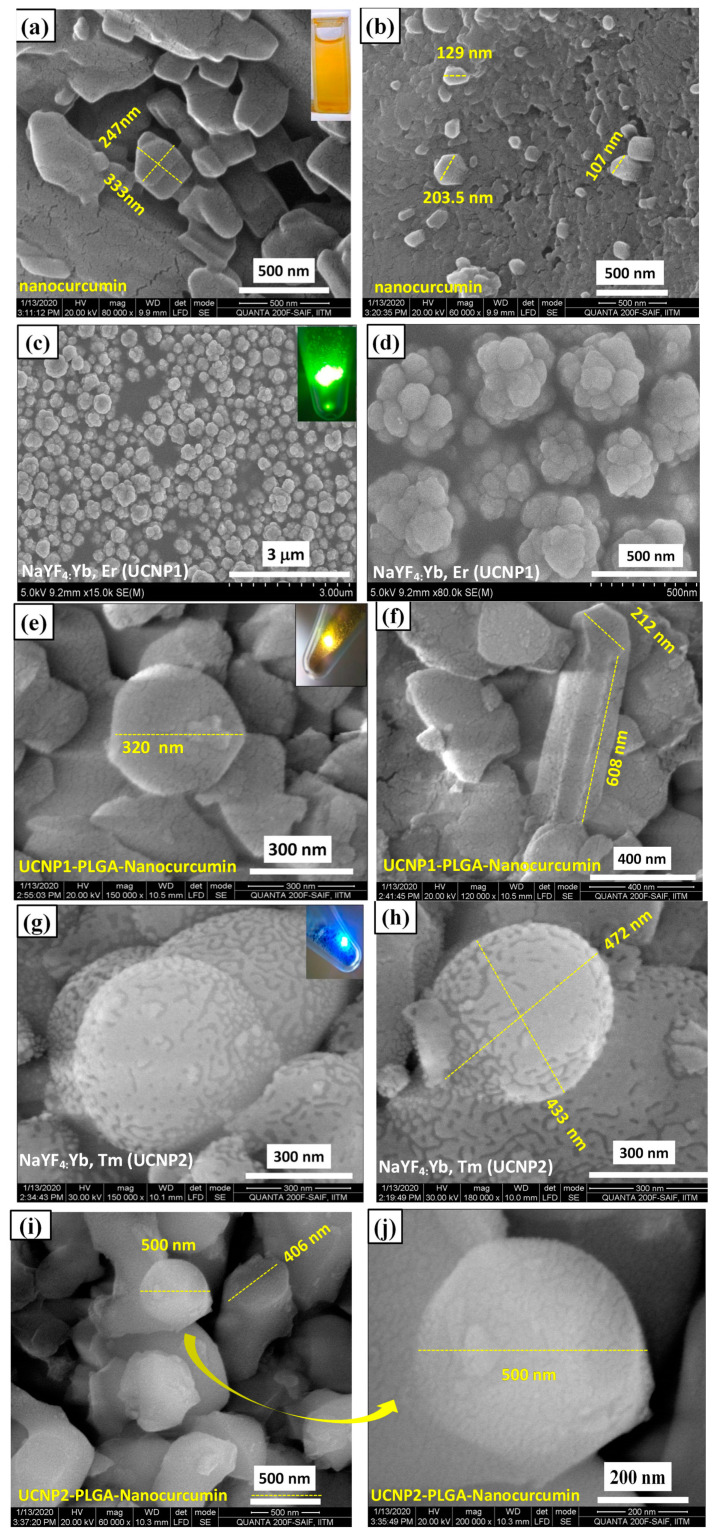
HRSEM images of nanocurcumin (**a**,**b**)**,** UCNP1 (**c**,**d**), UCNP1-PLGA-nanocur (**e**,**f**), UCNP2 (**g**,**h**), and UCNP2-PLGA-nanocur (**i**,**j**).

Figure 2a–j shows the HRSEM images of nanocurcumin (a,b), UCNP1(c,d), UCNP1-PLGA nanocur (e,f), UCNP2 (g,h) and UCNP2- PLGA-nanocur (i,j). Figure 2a,b represents the morphological behaviour of nanocurcumin and the particle size is 150–200 nm. Reverse microemulsion synthesized UCNP1 (Figure 2c,d) and UCNP2 (Figure 2g,h) are formed as spherical nanoclusters in size ~200–300 nm. Figure 2e,f shows the surface morphology of nanocurcumin-loaded UCNP1-PLGA, which shows nanospheres in size ~300 nm. Figure 2i,j shows that UCNP2-PLGA-nanocur with the size of ~350 nm. SEM-EDAX analysis was performed for finding the chemical species present in the UCNP1-PLGA-nanocur composite. The EDAX spectrum shown in Figure S3a–c explores the existence of Yb and Er dopant ions and host material elements Na, Y and F in the UCNP1-PLGA-nanocur composite. The detailed analysis on the EDAX spectrum is included in the supplementary information. Figure S4 displays the elemental mapping and distribution of the Na, Y, F, Yb, Er, C, and O. The upconversion nanoparticles are considered as attractive drug carriers because of their remarkable benefits of increased tumour-specific targeting ability and minimal side effects along with superior drug loading capability [29]. It is reported that the sphericity plays a major role in drug releasing properties [28,30] and particle size is an essential criterion in drug delivery [2]. The large-sized PLGA copolymer functionalized UCNP could effectively encapsulate the drug and increase drug loading [31]. The large and porous structured UCNP could promote drug loading ability [29,32]. To analyse the stability of UCNP and nanocurcumin, their zeta potential was measured by dynamic light scattering method and the results are given Figure S5 and Table S1.

### 3.3. X-ray Diffraction Analysis

**Figure 3:**
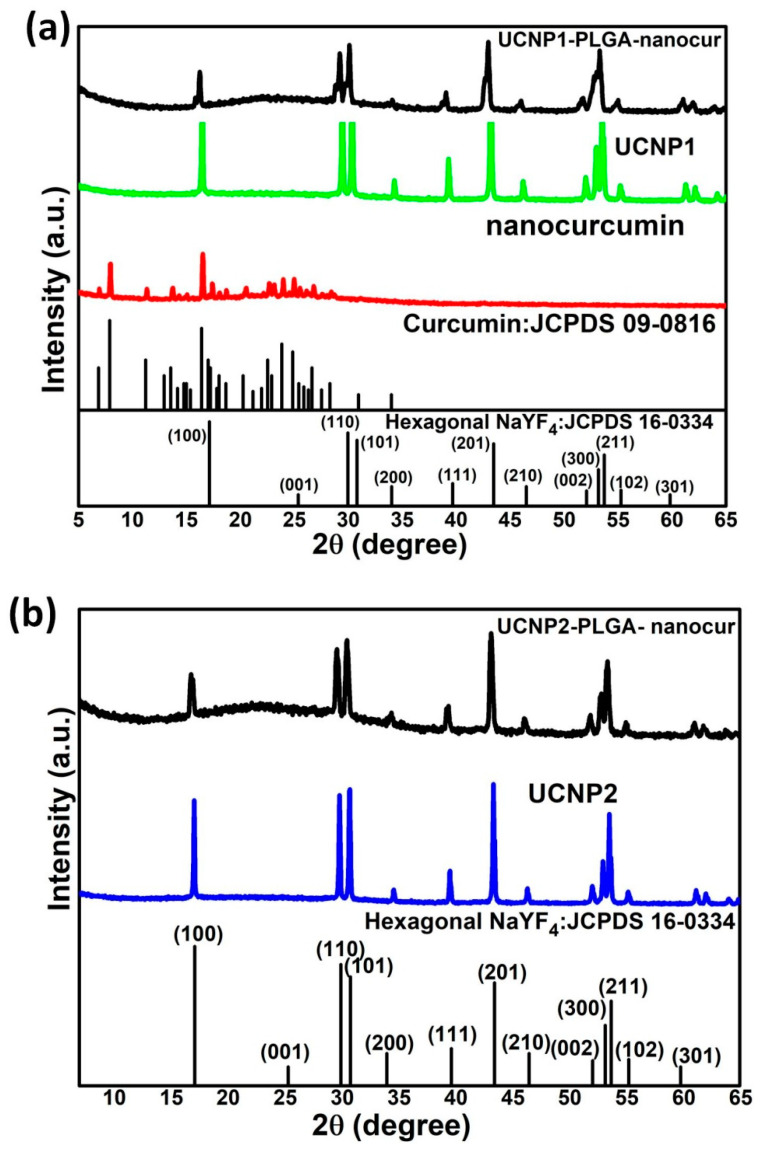
XRD patterns of (**a**) NaYF_4_:Yb,Er (UCNP1), nanocurcumin, UCNP1-PLGA-nanocur (**b**) NaYF_4_:Yb,Tm (UCNP2), and UCNP2-PLGA-nanocur.

At each stage of the preparation process, for the structural phase determination of UCNPs and drug encapsulated UCNPs, the X-ray powder diffraction was employed. Figure 3a reveals the XRD patterns of nanocurcumin, UCNP1 and UCNP1-PLGA-nanocur, respectively. Figure 3b displays the XRD spectrum of UCNP2 and UCNP2-PLGA-nanocur, respectively. The diffraction patterns of the synthesized nanocurcumin exhibits five distinctive peaks at 12.21, 17.13, 24.59, 25.58 and 26.23° in the two-theta range of 10 to 30°. The result is consistent with the JCPDS: 09-0816 as can be verified in Figure 3a. It implies the synthesized nanocurcumin is formed in crystalline form [33]. The diffraction peaks of reverse micro-emulsion synthesized UCNP1 and UCNP2 are matches well the JCPDS:16-0334 of hexagonal β-NaYF_4_. The hexagonal phase UCNP1 and UCNP2 are crystalline with the space group of p63/m [5]. The diffraction peaks of the UCNP1-PLGA-nanocur and UCNP2-PLGA-nanocur broaden with a decrement in their peak intensity compared to UCNP1 and UCNP2, and it can be identified at 29 and 30° corresponding to the (110) and (101) planes. The broad peak indicates the incorporation of nanocurcumin with UCNPs. It is also noticed that in the XRD pattern of UCNP1-PLGA-nanocur and UCNP2-PLGA-nanocur, the intense and sharp characteristic diffraction peaks related to nanocurcumin did not appear from 5 to 30°. The absence of nanocurcumin peaks is due to the dominated hexagonal structure of UCNPs in the UCNP1-PLGA-nanocur and UCNP2-PLGA-nanocur samples [14]. Even after the addition of PLGA and nanocurcumin drug loading, the hexagonal UCNP is stable. This is advantageous for biomedical applications. The lattice parameters and cell volume is given in Table 1, The diffraction pattern of nanocurcumin and PLGA encapsulated UCNPs nanoparticles is slightly broadened compared to UCNP1 and UCNP2 owing to the polymer. This result is comparable with the XRD pattern of PLGA 50:50 nanoparticles reported by Mahajan et al. [34].

### 3.4. FTIR Absorption Spectroscopy

**Figure 4:**
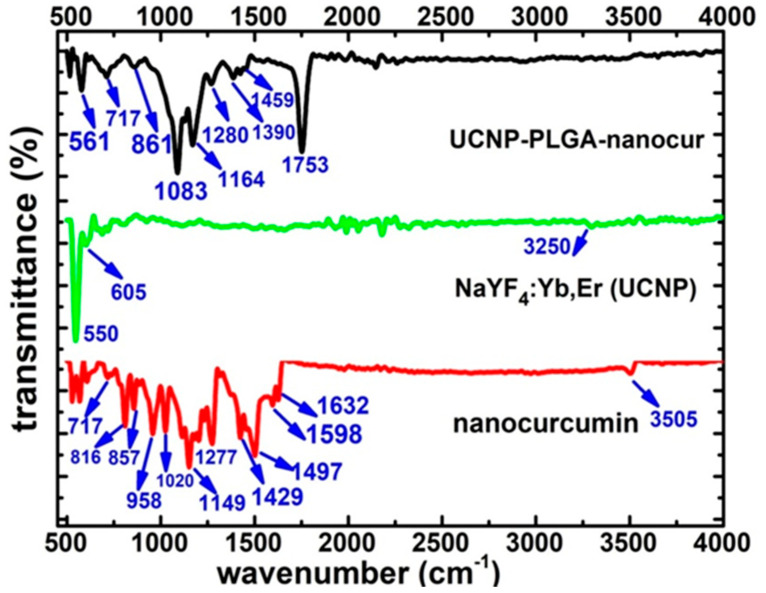
FTIR spectra of nanocurcumin, NaYF_4_:Yb,Er and UCNP-PLGA-nanocur.

The FTIR spectra of nanocurcumin, NaYF_4_:Yb,Er (UCNP) and UCNP-PLGA-nanocur samples are displayed in Figure 4. The nanocurcumin shows a weak peak at 717 cm^−1^ allocated for the C−H vibrations of the aromatic ring [14]. The other peaks at 816 cm^−1^ and 857 cm^−1^ correspond to the C-H bending [33]. The peak at 958 cm^−1^ is allotted for the benzoate trans-C−H vibration [14]. The other peak at 1020 cm^−1^ may be attributed for C−O−C groups [33] and peak at 1149 cm^−1^ is assigned to the functional group of C−H stretching [11]. Then peak at 1277 cm^−1^ represent the C−O stretching group and the peak at 1429 cm^−1^ corresponds to phenolic C−O stretching [35].The absorption peak seen at 1497 cm^−1^ is assigned to the C=O and C=C vibrations [36]. The strong absorption peak that arises from 1598 cm^−1^ can be denoted for the symmetric stretching vibrations about the aromatic ring (C=C ring) [17]. Another absorption peak noticed at 1632 cm^−1^ might be due to the (C=C) stretching [11]. The peak located around ~3505–3510 cm^−1^ expresses that there is presence of OH stretching in the glucose moiety in the nanocurcumin. The presences of all the characteristics peaks of nanocurcumin in the FTIR spectrum suggest that there is no chemical alteration or notable degradation of the drug during the solvent-antisolvent process [14,16,33,36].

NaYF_4_:Yb,Er (UCNP) exhibits a strong absorption peak around 550 cm^−1^ and small absorption peak at 605 cm^−1^ which corresponds to stretching vibrations of metal fluorides. An absorption peak observed at 3250 cm^−1^ represent the stretching vibration of the amine groups (–NH_2_) of CTAB (capping ligand) surfactant prepared by the reverse micro-emulsion method. Hence, the contribution of the amine surfactant moieties improves the biocompatibility and the hydrophilic property of the drug carrier [5].

Whereas, the FTIR spectrum of UCNP-PLGA-nanocur exhibits the existence of functional groups present in the nanocurcumin and UCNPs. Among these, the characteristic functional groups of nanocurcumin at 717, 861, and 1280 cm^−1^ could be identified from the drug incorporated sample. Particularly, the absorption peaks for the nanocurcumin around 861 and 1280 cm^−1^ experienced a mild shift to 857 and 1277 cm^−1^ in the drug loaded sample due to nano-encapsulation and indicates successful drug loading. Similarly, the identified metal fluoride functional group of UCNPs at 550 cm^−1^ also involved with slight shift to 561 cm^−1^ owing to the interaction between drug and UCNPs. In addition, C−O− stretch of C−O−H groups rose in 1083 cm^−1^ and also the absorption peak occurring at 1459 denoted for the C−O−H in plane bending of carboxylic acid (−COOH) [31]. It is to be noted that the new strong absorption peak arises from ~1753 cm^−1^, which might signify the characteristic C=O stretching of acid group due to the interaction of PLGA polymer [25,30]. The small absorption peaks at 1164 and 1280 cm^−1^ indicate the C−O stretching. The other absorption peaks found at 1390 and 1459 cm^−1^ are assigned to O−H bending vibrations and C-H bending, C=C stretching vibrations for the aromatic ring independently. It affirms that the nanocurcumin efficiently loaded with UCNPs via PLGA ligands attached on the surface of the nanocarrier. The PLGA functionalization with carboxylic groups could improve the binding affinity between the nanocurcumin drug and the UCNP nanocarrier, which will be useful for drug delivery applications [5].

### 3.5. Raman Spectroscopy

**Figure 5:**
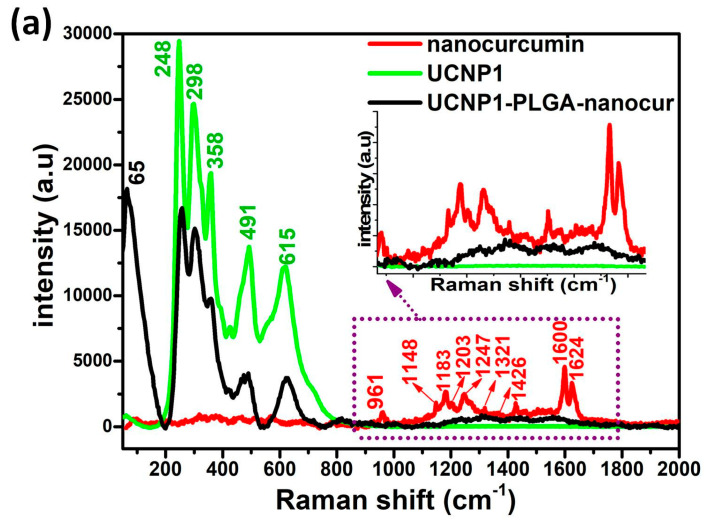

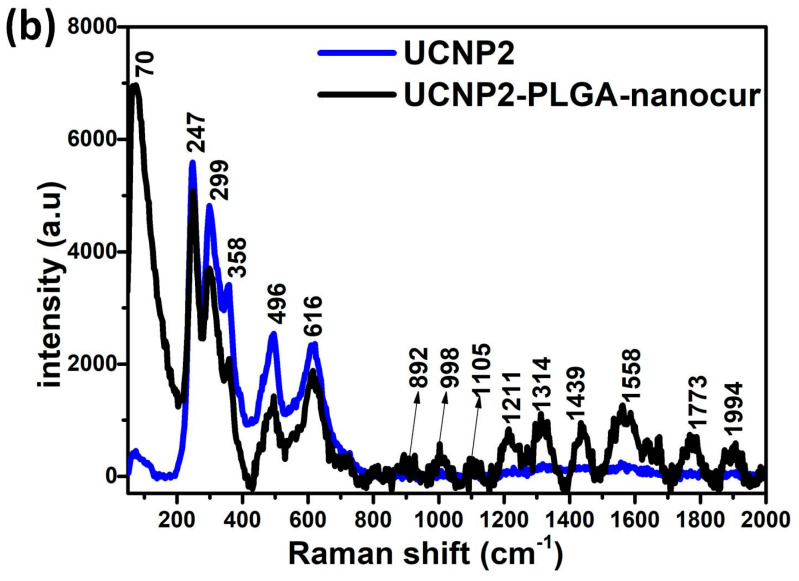
(**a**) Raman spectra of Nanocurcumin, NaYF_4_:Yb,Er (UCNP1), UCNP1-PLGA-nanocur (**b**) NaYF_4_:Yb,Tm (UCNP2), UCNP2-PLGA-nanocur composites.

Figure 5a,b shows the Raman spectra recorded for nanocurcumin, UCNP1, UCNP1-PLGA-nanocur and UCNP2, UCNP2-PLGA-nanocur. The Raman vibrational modes of the nanocurcumin are located from 950 cm^−1^ to 1700 cm^−1^ as shown in Figure 5a, and the results were in concurrence to the reported data [37]. The Raman spectra for the UCNP1 and UCNP2 materials display five main peaks originating from 200–650 cm-1 along with the two dominant peaks around 245–300 cm^−1^, which are the characteristic peaks of NaYF_4_ host lattice. Raman peaks located from the scale of 400 to 700 cm^−1^ might be denoted for the vibrational frequencies of Na-F. High phonon vibration modes above 1000 cm^−1^ are weakly present and almost absent in the UCNP1 and UCNP2 [38,39].

In the case of UCNP1-PLGA-nanocur and UCNP2-PLGA-nanocur, the spectrum displays Raman peaks corresponding to both nanocurcumin and UCNPs, which is an indication of drug loading. No Raman peak is observed above 1000 cm^−1^ in the UCNP1 and UCNP2. However, the inset of Figure 5a reveals UCNP1-PLGA-nanocur shows Raman modes (1000–1600 cm^−1^) due to the drug loading of nanocurcumin with the UCNPs. In addition, the major functional groups of UCNPs that lies from 200–800 cm^−1^ remains unaffected by the incorporation of nanocurcumin except a slight decrement in intensity. Similar results are obtained for UCNP2-PLGA-nanocur displayed in Figure 5b. The Raman spectral result indicates the nanocurcumin and UCNP nanocarrier is conjugated by the PLGA polymer in the UCNP1-PLGA-nanocur and UCNP2-PLGA-nanocur composites, which could be useful for biomedical applications.

### 3.6. NIR to Visible Upconversion Emission Characteristics of NaYF_4_:Yb,Er/Tm

**Figure 6:**
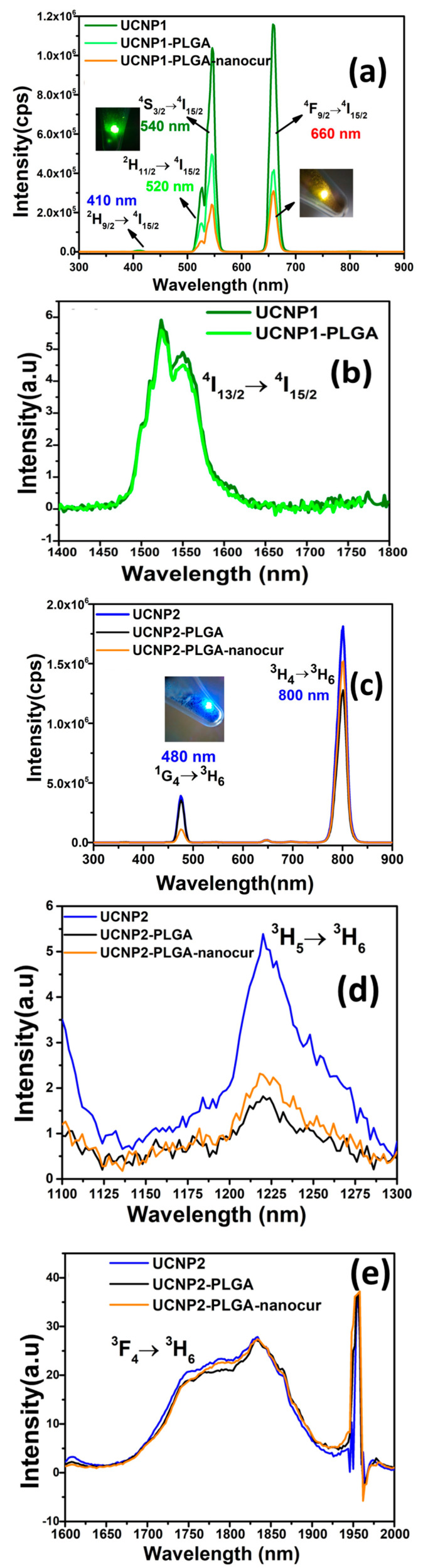
(**a**) UCL spectra of UCNP1, UCNP1-PLGA and UCNP1-PLGA-nanocur, (**b**) fluorescence spectra of UCNP1, UCNP1-PLGA, (**c**) UCL spectra of UCNP2, UCNP2-PLGA and UCNP2-PLGA-nanocur (**d**,**e**) fluorescence spectra of UCNP2, UCNP2-PLGA and UCNP2-PLGA-nanocur, respectively.

Figure 6a–e shows the NIR to visible upconversion emission and NIR to NIR down conversion emission spectra of NaYF_4_:Yb,Er/Tm UCNPs, PLGA-UCNPs and nanocurcumin incorporated PLGA-UCNPs. Figure 6a exhibits the upconversion luminescence (UCL) spectra of hexagonal UCNP1, UCNP1-PLGA and UCNP1-PLGA-nanocur under 980 nm excitation. It exhibits emission at 410, 520, 540, and 660 nm, which correspond to transitions arising out of ^2^H_9/2_, ^2^H_11/2_, ^4^F_7/2_, ^4^S_3/2_ excited levels towards the ^4^I_15/2_ ground level of Er^3+^ ion [40,41]. Hexagonal UCNP1 displays the upconverted intense green and red emission. Xilin Bai et al. demonstrated that strong green emission of UCNPs shows great bioimaging ability and reported the UC red emission is useful for cell therapy [42]. The UCNP1-PLGA provides similar UC emission characteristics, but it shows less intense emission due to the carboxylic functional groups of PLGA [43], which could improve the drug internalization capacity. Nevertheless, the UC emission in PLGA-UCNP could be sufficient enough for bioimaging. A decrement in the UC emission is noticed in the UCNP1-PLGA-nanocur composite due to the presence of organic moiety [44]. However, UC emission of UCNP1-PLGA-nanocur shown in Figure 6a indicate it has considerable spectral intensity of ~2.5 × 10^5^ cps for green emission and ~3 × 10^5^ cps for red emission, which may be suitable for cancer imaging and treatment under the action of NIR laser radiation. The upconverted yellow emission was observed from the UCNP1-PLGA-nanocur (inset of Figure 6a) under the influence of 200 mw 980 nm NIR diode laser pointer. The UC yellow emission in UCNP1-PLGA-nanocur is for the combination of green and intense red emissions. It implies the nanocurcumin is encapsulated over the UCNPs through PLGA conjugation [30,45] and UC emission in the nanocurcumin drug loaded PLGA-UCNP could play a certain role in cancer bioimaging and therapeutics. Figure 6b shows the NIR-to-NIR down conversion fluorescence emission at 1400–1800 nm for the UCNP1 and UCNP1-PLGA nanoparticles.

Figure 6c displays the upconversion luminescence emission spectra of UCNP2, UCNP2-PLGA, and UCNP2-PLGA-nanocur composite. All the three samples exhibit weak emission at 480 nm and intense NIR emission at 800 nm, which are, respectively, assigned to ^1^G_4_ → ^3^H_6_ and ^3^H_4_ → ^3^H_6_ transitions of Tm^3+^ ion [40,41]. The reduction in the peak intensity after PLGA conjugation and nanocurcumin loading are owing to the polymer moiety. The UCNP2-PLGA-nanocur exhibiting blue emission and intense NIR to NIR upconversion emission is a novel result for biological applications. As NIR emission of Tm^3+^ falls interior in the biological window 700–900 nm, it could lead to low scattering and high penetration in bio tissues.

### 3.7. NIR to NIR Downconversion Emission Characteristics

Figure 6b shows the NIR-to-NIR down conversion fluorescence spectra of UCNP1 and UCNP1-PLGA nanoparticles taken in the range of 1400–1800 nm. The emission spectra around 1550 nm is for the ^4^I_13/2_ → ^4^I_15/2_ energy level transition in Er^3+^ ions. Figure 6d shows the NIR-to-NIR down conversion fluorescence spectra of UCNP2, UCNP2-PLGA and UCNP2-PLGA-nanocur from 1100–1300 nm. The infrared emission spectrum centred at 1225 nm corresponds to ^3^H_5_ → ^3^H_6_ transition of Tm^3+^ ion. Figure 6e reveals the downconversion fluorescence spectra of UCNP2, UCNP2-PLGA and UCNP2-PLGA-nanocur from 1600–2000 nm. The infrared emission spectrum centred at 1800 nm corresponds to ^3^F_4_ → ^3^H_6_ transition of Tm^3+^ ion.

### 3.8. FRET Mechanism between UCNPs and Nanocurcumin for PDT

Based on the literature evidence, the energy transfer mechanism between the UCNP and nanocurcumin for photodynamic therapy (PDT) applications could be explained [46]. It is well known that curcumin is not only used as a natural drug to treat various diseases, but it also used as a photosensitizer for PDT [47]. The synthesized nanocurcumin has optical absorption in the UV (270 and 350 nm) and visible (~400 to 450) spectral range (Figure S1, discussed in the supplementary section). By exploiting the broad optical absorption behaviour of curcumin, one can use it as photosensitizer molecule for PDT by blue light activation [48,49,50] by blue LED or laser sources. However, weak point is that it does not have NIR absorption capability, and hence it is not suitable for NIR light induced PDT applications. When nanocurcumin is functionalized with NaYF_4_:Yb,Tm nanoparticle, under the influence of 980 nm diode laser excitation (Figure 6c), the curcumin can be activated by the upconverted light at 450 to 500 nm by resonant energy transfer from the Thulium donor ion of UCNPs to the acceptor nanocurcumin photosensitizer. It has been reported that the Förster resonant energy transfer between the UCNP and curcumin molecule [51] could provide dual activation in the UCNP-nanocurcumin nanocomplex, generate reactive oxygen species (ROS) and kill the cancer cells. Therefore, UCNP-Nanocurcumin could be served as a potential nanocomplex for PDT compared to the bare nanocurcumin [52].

### 3.9. In Vitro Cytotoxicity Studies

**Figure 7:**
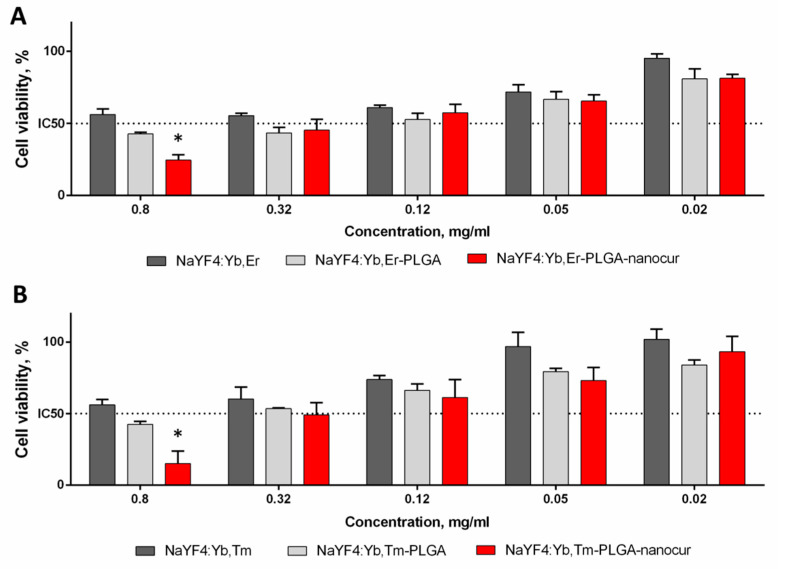
Viability of rat glioma C6 cells after 48 h of incubation with NaYF_4_:Yb,Er-based (**A**) and NaYF_4_:Yb,Tm-based (**B**) nanoparticles. MTT assay, the viability of intact cells was taken as 100%. * *p* < 0.05 in comparison to blank nanoparticles in Mann–Whitney U test (non-parametric, two-tailed).

The in vitro cytotoxicity of the bare drug-free UCNPs and nanocur-loaded UCNPs was evaluated using rat glioma C6 cells (Figure 7). We used glioma cells since the potential of curcumin in the glioma and glioblastoma treatment has been demonstrated earlier [53,54]. The blank NaYF_4_:Yb,Er and NaYF_4_:Yb,Tm nanoparticles demonstrated dose-dependent cytotoxicity with slight cell growth inhibition at 0.05 mg/mL and higher; however, even at 0.8 mg/mL cell viability was above 50% that confirmed the suitability of developed UCNPs for biomedical applications. The NaYF_4_:Yb,Er-PLGA-nanocur and NaYF_4_:Yb,Tm-PLGA-nanocur found to more toxic to cells, and cell viability at 0.8 mg/mL was 24 ± 4% (*p* < 0.05) and 19 ± 6% (*p* < 0.05), respectively. It should be noted, that at lower concentrations the toxicity of nanocur-loaded and blank UCNPs was similar that could be explained with prolong drug release and comparatively low nanocurcumin toxicity. The curcumin loaded porous silica tested in HT-29 and NCM460 cells. The cell viability was reported to be 50% at a low concentration of 50 µg/mL or 0.05 mg/mL [55], and at this concentration the present UCNP-PLGA-nanocurcumin showed more than 60% cell viability. The nanocurcumin loaded UCNP-PLGA nanocomplexes showed moderate cytotoxicity (Figure 7) against the rat glioma C6 cells compared to drug free UCNPs. It may give good cell viability with other cancer cell lines, but it has to be investigated in detail. By carefully controlling the particle size and solubility of nanocurcumin, its physico-chemical properties could be improved to make it useful for anti-cancer application. In addition to the in vitro cell viability, in order to utilize the UCNP-Nanocurcumin complexes for PDT applications, it is important to examine the phototoxicity of UCNP under illumination of NIR light activation. It is planned to investigate the phototoxicity of present UCNP-Nanocurcumin complexes and present as an extension of the current work. The Russian researchers Minorova et al. and Khayduko et al. investigated the phototoxicity effect of specially designed NaYF_4_:YbEr and NaYF_4_:YbTm nanoparticles under the 975 nm NIR diode laser illumination and demonstrated a new approach on PDT by the UCNP mediated UV and UV-blue light excitation mechanism to generate ROS for killing the cancer cells [56,57].

### 3.10. Tumor Spheroids Model

The toxicity of UCNPs was also evaluated in tumour spheroids model. Tumour spheroids are three-dimensional cell aggregates that mimic some features of tumours in vivo, including cell–cell and cell–matrix interactions, gradients, and higher drug resistance, so tumour spheroids could be discussed as an advanced in vitro model of in vivo tumours [58,59].

**Figure 8:**
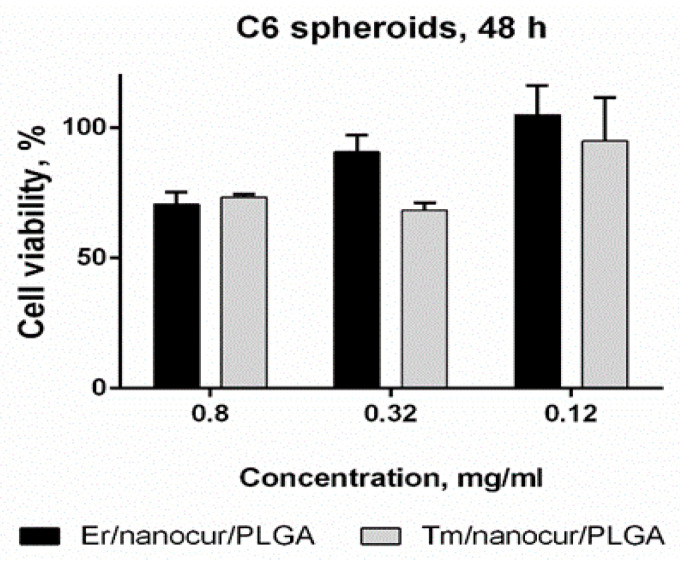
Viability of C6 cells in spheroids after 48 h of incubation with NaYF_4_:Yb,Er-PLGA-nanocur and NaYF_4_:Yb,Tm-PLGA-nanocur. MTS assay, the viability of intact cells was taken as 100%.

**Figure 9:**
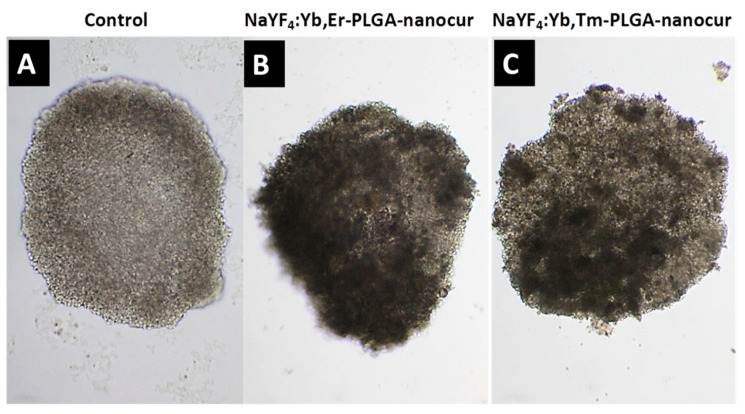
Micrographs of rat glioma C6 spheroids incubated with 0.4 mg/mL UCNPs for 48 h, lens 10×. Spheroids represent the 3D cell culture model similar to tumors in vivo. The outer cell layer is damaged in case of treated spheroids (**B**,**C**) in comparison to the control intact spheroid (**A**).

**Figure 10:**
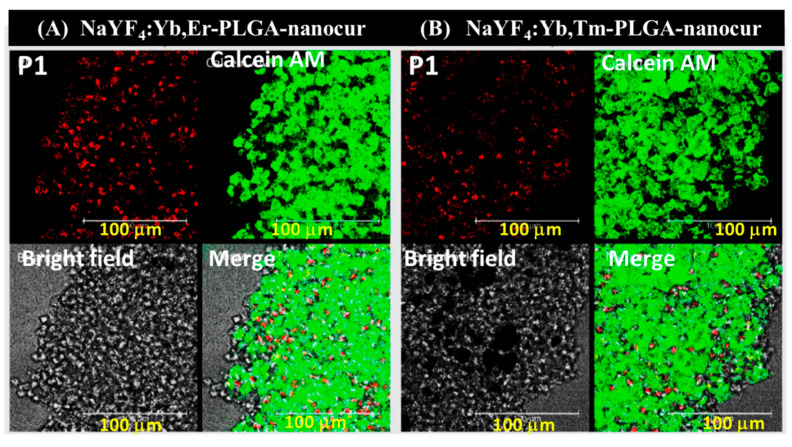
Confocal fluorescent microscopy micrographs of rat glioma C6 spheroids incubated with 0.4 mg/mL UCNPs for 48 h, NaYF_4_:YbEr PLGA nanocur (**A**) and NaYF_4_:YbTm PLGA nanocur (**B**) The spheroids were stained with propidium iodide (dead cells, in red) and calcein AM (alive cells, in green), scale bar is 100 μm.

Indeed, C6 cells in spheroids were more resistant to treatment with NaYF_4_:Yb,Er-PLGA-nanocur and NaYF_4_:Yb,Tm-PLGA-nanocur in comparison to monolayer culture with viability of 70 ± 6% and 74 ± 3% at 0.8 mg/mL, respectively (Figure 8). The disruption of the outer cell layer of spheroids within 48 h treatment in comparison to control intact spheroid (A) is demonstrated on (B, C) in Figure 9. By using the confocal fluorescent microscopy, the cell viability in glioma C6 spheroids was visualized with calcein AM (alive cells) and propidium iodide (dead cells) staining (Figure 10). Propidium iodide staining (in red) of cell nucleuses confirms partial cell death in spheroids under NaYF_4_:Yb,Er-PLGA-nanocur (A) and NaYF_4_:Yb,Tm-PLGA-nanocur (B) treatment.

### 3.11. In Vitro Distribution of UCNPs in Rat Glioma Cells and Imaging Studies

**Figure 11:**
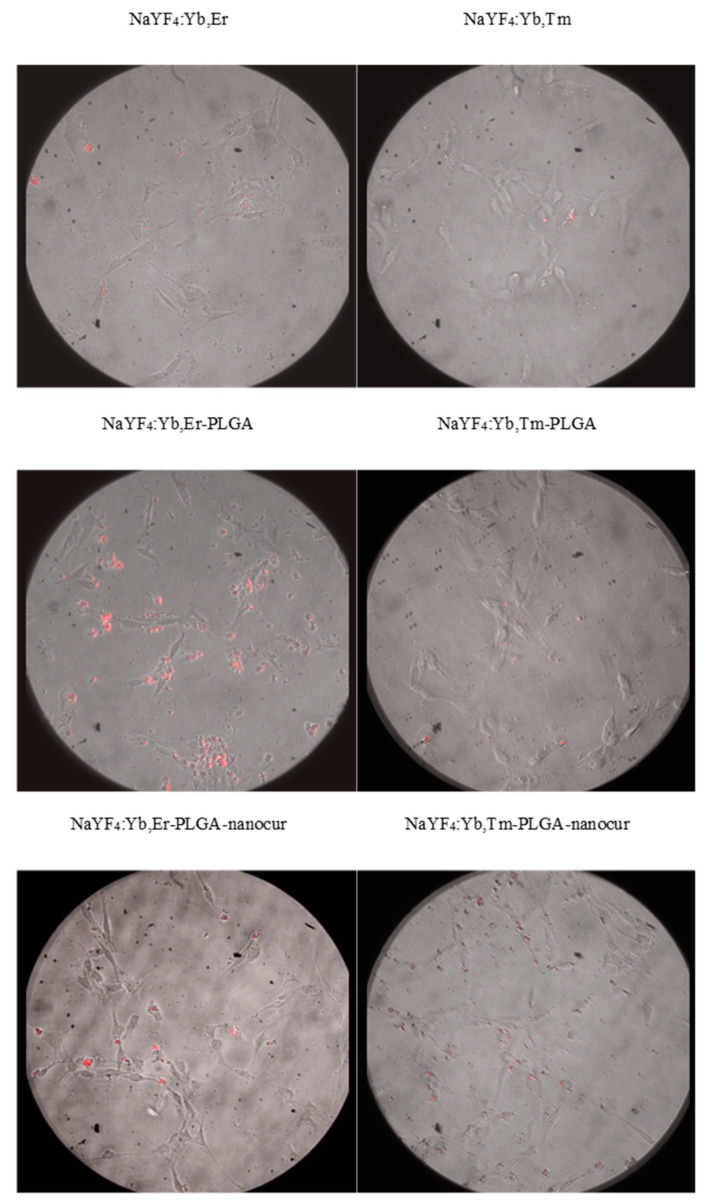
Micrographs of rat C6 glioma cells in an upconverting luminescence microscope: UCNPs concentration 0.05 mg/mL, 30 min of incubation. The upconversion signal is in red. Lens 10×.

**Figure 12:**
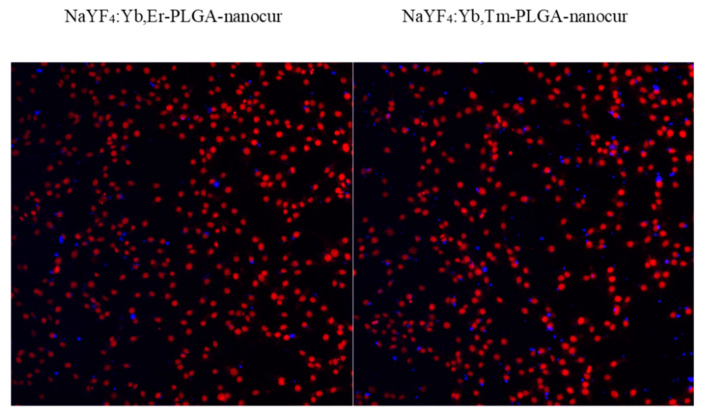
Micrographs of rat C6 glioma cells under a fluorescence microscope: UCNPs concentration 0.05 mg/mL, 30 min of incubation. Cell nuclei are marked in red, the fluorescence of nanocurcumin is marked in blue. Excitation of nanocurcumin was induced by irradiation at 488 nm. Lens 10×.

**Figure 13:**
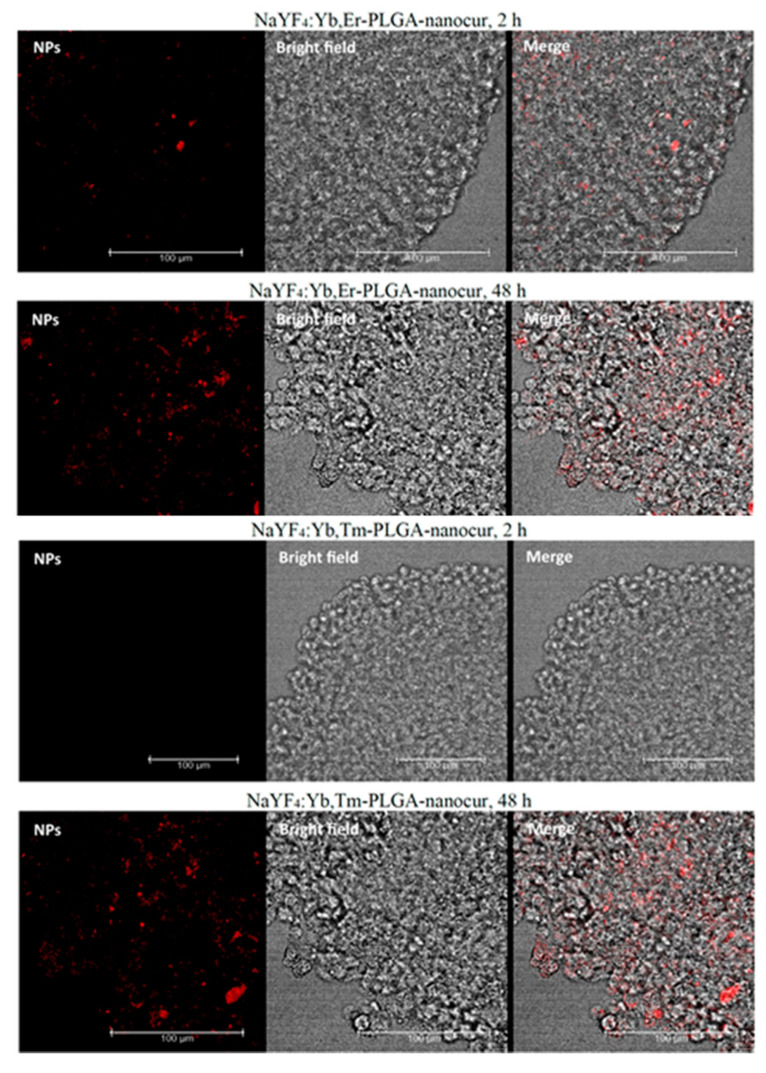
Micrographs of rat glioma C6 spheroids, 0.4 mg/mL UCNPs, 2 h and 48 h incubation, confocal fluorescent microscopy. The fluorescence of nanocurcumin is indicated in red, 488 nm excitation. Scale bar is 100 μm.

We evaluated the in vitro distribution of UCNPs in rat C6 Glioma cells using anti-Stokes photoluminescence microscopy and confocal fluorescence microscopy. It was demonstrated that UCNPs were able to accumulate in cells within 1 h, and this could be visualized both in anti-Stokes photoluminescence (Figure 11) and fluorescence (Figure 12) mode. Both approaches provide similar UCNPs distribution in cells, but anti-Stokes photoluminescence provides higher signal-to-noise ratio due to the absence of background signal. It should be noted that PLGA-coated and PLGA-nanocur-coated UCNPs better accumulated in cells that could be explained with cell-particles interactions in case of PLGA modification (Figure 11). This correlates with MTT assay data that were discussed above. Since the penetration into solid tumours is one of the limitations for nano-based formulations, we additionally evaluated the accumulation of UCNPs in tumour spheroids. We demonstrated the accumulation of UCNPs in spheroids after 48 h incubation, but not after 2 h (Figure 13). The penetration depth is at least 100 µm.

### 3.12. In Vivo Distribution of UCNPs and Small Animal Imaging Studies

**Figure 14:**
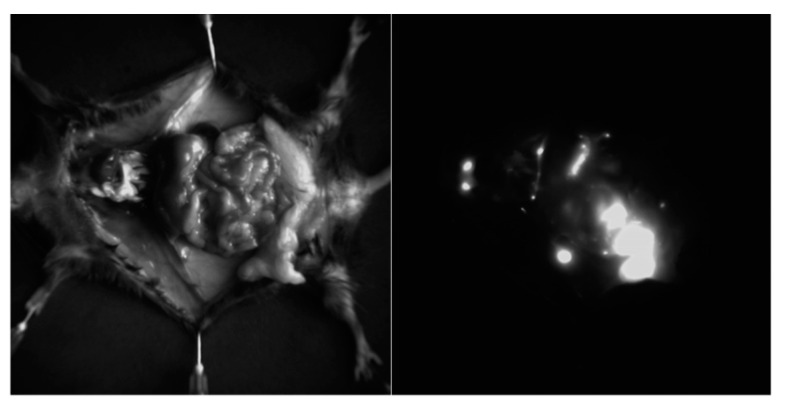
Bright-field image of the mouse organocomplex (**left**) and epiluminescent image of the mouse organocomplex marked with NaYF_4_:Yb,Tm -PLGA-nanocur (**right**), intravenous injection of 0.75 mg UCNPs, 4 h after injection.

**Figure 15:**
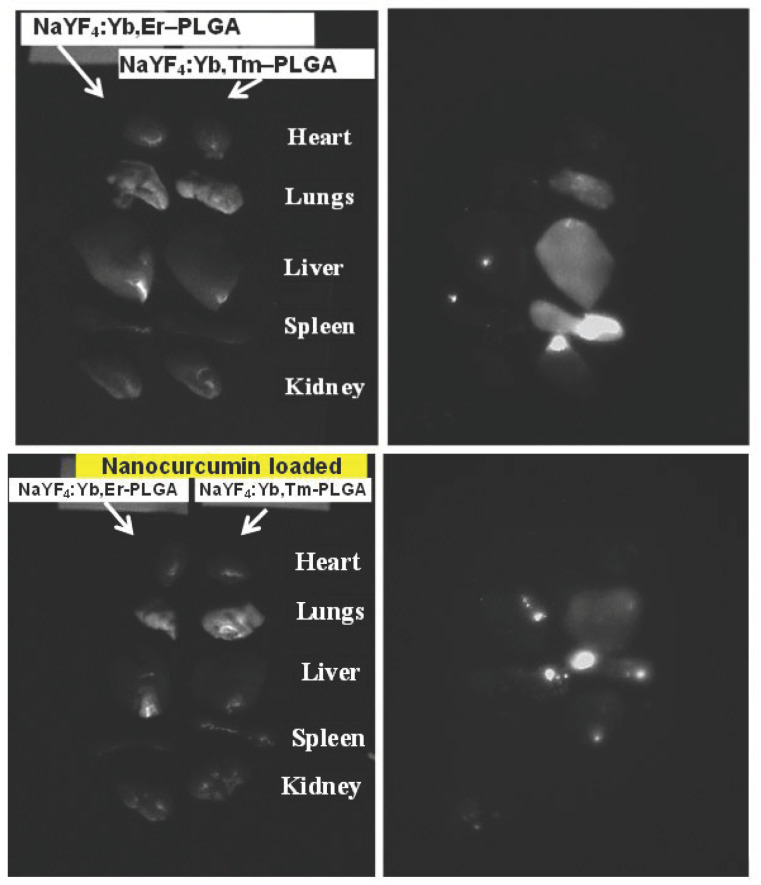
Bright-field image of the ex vivo organs (**left**) and epiluminescent image of the mouse organocomplex (**right**). Intravenous injection of 0.75 mg UCNPs, 4 h after injection. (Upper row-NaYF_4_:Yb,Er-PLGA and NaYF_4_:Yb,Tm-PLGA, lower row-NaYF_4_:Yb,Er-PLGA-nanocur and NaYF_4_:Yb,Tm-PLGA-nanocur).

We evaluated the biodistribution of UCNPs in vivo using DVS-02 small animals imaging system. We found that UCNPs accumulated in the liver, lungs, intestines, and spleen in 4 h after administration (Figure 14 and Figure 15), which corresponds to the common biodistribution pattern of nanoparticles after systemic administration. A significant amount of UCNPs in the intestine confirms their ability to be excreted from the body. Indeed, the UCNPs signal decreased significantly after 24 h post-injection. It should be noted that NaYF_4_:Yb,Tm showed a brighter luminescent signal compared to NaYF_4_:Yb,Er, which can be explained by a higher quantum yield. At the same time, a slight decrease of the signal was found in the NaYF_4_:Yb,Tm-PLGA-nanocur sample in comparison with NaYF_4_:Yb,Tm-PLGA (Figure 15). Lewis lung cancer was used as a mouse tumour model. We demonstrated that in the case of peritumoral administration, UCNPs were detected in the tumour site for a long time (at least 24 h), which indicates the ability of UCNPs to persist in the tumour tissue. 

## 4. Conclusions

In summary, we demonstrated assembling nanocurcumin with UCNPs via introducing an intermediate stage of UCNP coating with PLGA endowed the as-synthesised nanocomplexes optical contrast in the near-infrared transparency window. NaYF_4_:Yb,Er-PLGA-nanocurcumin and NaYF_4_:Yb,Tm-PLGA-nanocurmin, respectively, exhibited upconversion emission at 520, 540, 660 nm and 480 and 800 nm spectral bands at 980 nm diode laser excitation. PLGA functionalized NaYF_4_:Yb,Er/Tm and nanocurcumin drug loaded PLGA-UCNPs showed 60–80% cell viability at 0.12–0.02 mg/mL in the rat C6 glioma cell medium. In vitro distribution of UCNPs in rat C6 glioma cells and imaging studies demonstrated the accumulation of UCNPs in the cancer spheroids. Peritumoral administration of UCNP-PLGA-nanocur to Lewis lung cancer bearing animal models rendered the tumour lesion optical contrast, which persisted for at least 24 h. This enabled in vivo and ex vivo study of UCNP-PLGA-nanocur biodistribution in organs, and showed accumulation in the liver and lungs. Our studies demonstrated promise of nanocurcumin-loaded upconversion nanoparticles for theranostics applications.

## Figures and Tables

**Table 1:** The lattice parameters of UCNP and the UCNP-PLGA-nanocurcumin complexes.

Sample	Crystal Structure	Lattice Parameters (Å)	Volume (Å^3^)
UCNP1	hexagonal NaYF_4_	a = 5.9404; c = 3.5063	107.508
UCNP1-PLGA-nanocur	hexagonal NaYF_4_	a = 6.015; c = 3.5120	110.041
UCNP2	hexagonal NaYF_4_	a = 5.9806; c = 3.5113	108.764
UCNP2-PLGAnanocur	hexagonal NaYF_4_	a = 6.0495; c = 3.5144	111.383
JCPDS No.16-0334	hexagonal NaYF_4_	a = 5.96; c = 3.51	107.974

## Supplementary Materials

The following are available online at https://www.mdpi.com/article/10.3390/nano11092234/s1, Figure S1: (a) UV-VIS absorption spectra (b) photoluminescence spectra of raw curcumin and nanocurcumin; Figure S2: Thermal analysis of nanocurcumin, (a) DTA and (b) TGA profile; Figure S3(a): EDAX survey spectrum of NaYF4:Yb,Er-PLGA-Nanocurcumin complex; Figure S3(b): EDAX spectrum in the range 0 to 3 keV shows strong peaks for the elements F, Y, and Na ion and less intense peaks from 1.4 and 2 kev related to M-shell X-ray emission energy lines for dopant Yb and Er ions. The organic species C, N, O are from PLGA polymer and curcumin drug,; Figure S3(c): EDAX spectrum in the range 6 to 10 keV shows very weak peaks for Yb and Er due to their L-shell X-ray emission energy lines; Figure S4: EDAX elemental mapping of Na, Y, F, C, O, Er, Yb elements in the NaYF4:Yb,Er-PLGA-Nanocur complex; Table S1: Zeta potential values of UCNP-Nanocurcumin complexes; Figure S5: Zeta potential profiles of the nanocurcumin, UCNP1 (NaYF4:Yb,Er), UCNP1-PLGA-nanocurcumin, UCNP2 (NaYF4:Yb,Tm) and UCNP2-PLGA-nanocurcumin complex (taken in aqueous solution at pH 7).
