# Supplementary material for: Nanocurcumin-Loaded UCNPs for Cancer Theranostics: Physicochemical Properties, In Vitro Toxicity, and In Vivo Imaging Studies
Source: Nanomaterials (Basel). 2021 Aug 29;11(9):2234. doi: 10.3390/nano11092234 (PMC8471946; doi:10.3390/nano11092234)
Supplement: Supplementary file 1 [file nanomaterials-11-02234-s001.zip › nanomaterials-1331976-Supplementary Materials (new).pdf]

nanomaterials

Supplementary Information

Nanocurcumin-Loaded UCNPs for Cancer Theranostics: Physicochemical Properties, In Vitro Toxicity, and In Vivo Imaging Studies

Anbharasi Lakshmanan [1], Roman A. Akasov [2,3,4,*], Natalya V. Sholina [2,3], Polina A. Demina [4], Alla N. Generalova [3,4], Ajithkumar Gangadharan [5,6], Dhiraj K. Sardar [5,6], Krishna Bharat Lankamsetty [7], Dmitry A. Khochenkov [8,9], Evgeny V. Khaydukov [2,3], Sergey V. Gudkov [10,11], Manonmani Jayaraman [12] and Senthilselvan Jayaraman [1,*]

1 Department of Nuclear Physics, Guindy Campus, University of Madras, Chennai 600025, Tamil Nadu, India; anbhulaksh118@gmail.com
2 I M Sechenov First Moscow State Medical University, 119991 Moscow, Russia; sholinanv@gmail.com (N.V.S.); khaydukov@mail.ru (E.V.K.)
3 Federal Scientific Research Center, "Crystallography and Photonics", Russian Academy of Sciences, Leninskiy Prospekt 59, 119333 Moscow, Russia; angeneralova@gmail.com
4 Shemyakin-Ovchinnikov Institute of Bioorganic Chemistry, RAS, 117997 Moscow, Russia; polidemina1207@yandex.ru
5 Department of Physics and Astronomy, University of Texas at San Antonio, San Antonio, TX 78249, USA; akgsh2005@gmail.com (A.G.); Dhiraj.Sardar@utsa.edu (D.K.S.)
6 Department of Natural Sciences, Texas Agriculture and Mechanical University, One University Way, San Antonio, TX 78224, USA
7 Federal State Budgetary Scientific Institution "Federal Scientific Agroengineering Center VIM" (FSAC VIM), 109428 Moscow, Russia; krish.agudguy@gmail.com
8 FSBI "N.N. Blokhin National Medical Research Center for Oncology", Ministry of Health of the Russian Federation, Kashirskoe Shosse 24, 115478 Moscow, Russia; khochenkov@gmail.com
9 Medicinal Chemistry Center, Togliatti State University, Belorusskaya Str.14, 445020 Togliatti, Russia
10 Biophotonics Center, Prokhorov General Physics Institute of the Russian Academy of Sciences, 38 Vavilova St., 119991 Moscow, Russia; s_makariy@rambler.ru
11 Department of Closed Artificial Agroecosystems for Crop Production, Federal State Budgetary Scientific Institution "Federal Scientific Agroengineering Center VIM" (FSAC VIM), 5 First Institutskiy pr-d, 109428 Moscow, Russia
12 Department of Chemistry, Quaid-E-Millath Government College for Women, Chennai 600002, Tamil Nadu, India; jmanon32@gmail.com
* Correspondence: roman.akasov@gmail.com (R.A.A.); jsselvan@hotmail.com or jsselvan16@gmail.com (S.J.)

Highlights of the main manuscript

- Microemulsion synthesis and upconversion studies of UCNP-PLGA-nanocurcumin
- $NaYF_4$: Yb,Er-PLGA-nanocur provide green and yellow emission at 980 nm laser excitation
- $NaYF_4$: Yb,Tm-PLGA-nanocur give blue & NIR-NIR emission suitable for in vivo imaging
- UCNP-PLGA-nanocur incubated with rat glioblastoma cancer cells reveal mild cytotoxicity
- In vivo and ex vivo of UCNP-PLGA-nanocur with Lewis lung cancer mouse tumor model

1. Optical Absorption and Luminescence Spectral Studies of Nanocurcumin

The optical absorption and luminescence properties of the as-received raw curcumin and synthesized nanocurcumin were investigated with the UV-VIS spectrometer and spectrofluorometer. The curcumin samples were dispersed in water and unfiltered

aqueous solution was used. The UV-VIS spectrum of the raw curcumin (Figure S1a) has no characteristic absorbance peak at 420–450 nm [1,2], instead it shows a broad curve from 350 to 550 nm. Relatively, nanocurcumin showed absorptions at 270 nm, 350 nm and 425 nm due to its reduction in the particle size and aqueous solubility and [3]. The improved solubility of nanocurcumin could be useful for drug delivery application [4,5]. Photoluminescence property of curcumin and nanocurcumin was studied at an excitation wavelength of 425 nm. The raw curcumin exhibits weak luminescence (Figure S1b) owing to its poor optical absorption, shows almost a flat-profile varies from 350 to 550 nm and its maxima located at 567 nm. But, the nanocurcumin shows ten times enhanced luminescence compared to raw curcumin. The nanocurcumin emission shows a broad spectrum, which ranges from 500 to 650 nm and its maxima centered at 567 nm. A high hydrophilicity of nanocurcumin is the reason for the increased light absorption and emission. The nanocurcumin attains a blue shift in the fluorescence spectrum, due to particle size reduction, which is favorable to enhance oral intake for drug delivery and biological applications [5]. Hence, the solvent antisolvent synthesis approach has improved the hydrophilicity of nanocurcumin which can make it as good photo-sensitizer for drug loading process.

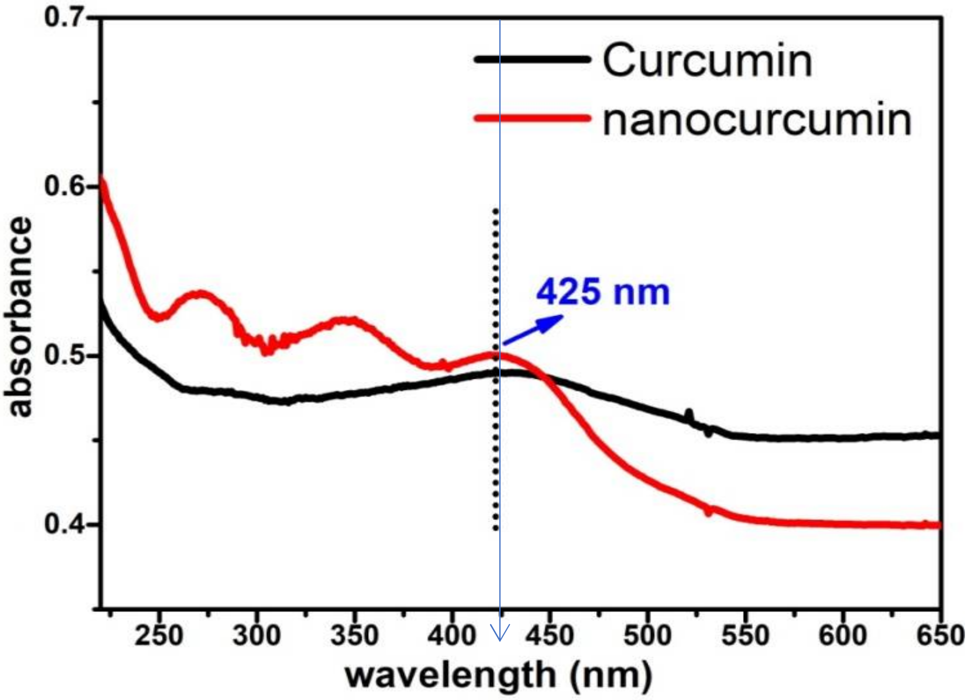

(a)

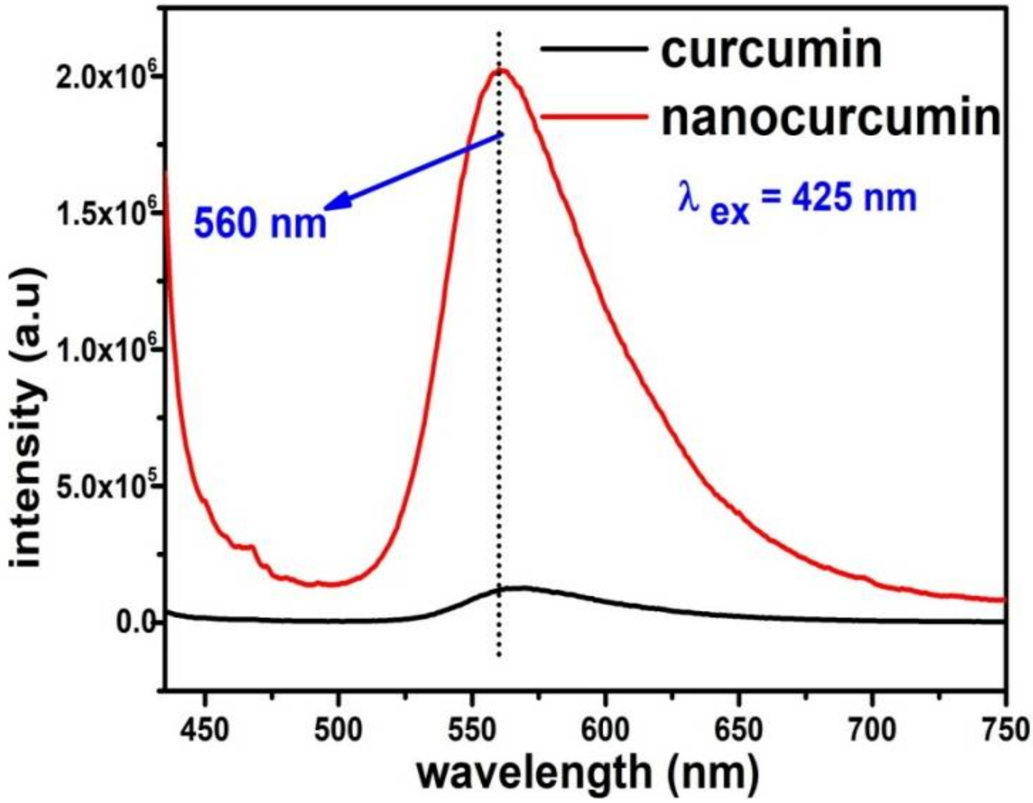

(b)

Figure S1. (**a**) UV-VIS absorption spectra (**b**) photoluminescence spectra of raw curcumin and nanocurcumin.

2. Thermal analysis

Thermal properties of the as-prepared nanocurcumin samples were studied using differential thermal analysis (DTA) and thermo gravimetric analysis (TGA). The DTA curve of nanocurcumin is shown in Figure S2a, which reveals an endothermic peak at 180 °C that related to the melting point of curcumin [6]. The TG curve of nanocurcumin is shown in Figure S2b, which shows the weight loss from 220 to 450 °C due to the chemical reduction curcumin molecule. It indicates nanocurcumin is stable up to 100 °C, so nanocurcumin was functionalized with UCNP-PLGA below 100 °C [7] to avoid decomposition and hence it was not heat-treated further.

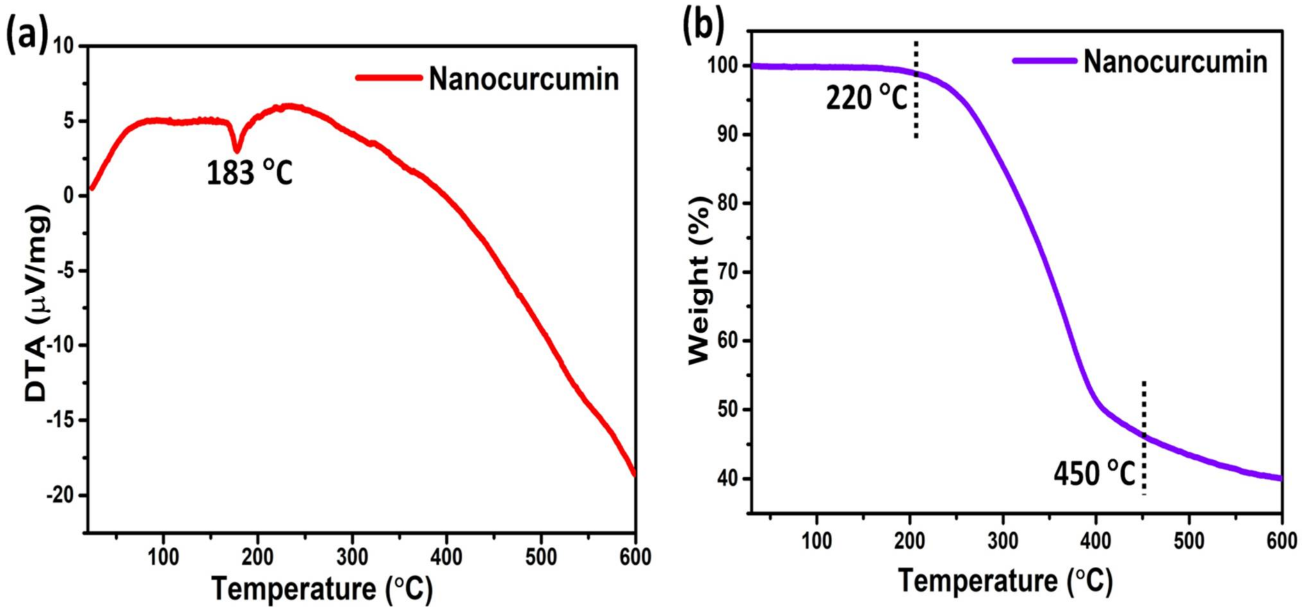

Figure S2. Thermal analysis of nanocurcumin, (**a**) DTA and (**b**) TGA profile.

3. EDAX and Elemental Mapping of $NaYF_4$:Yb,Er (UCNP1)-PLGA nanocur

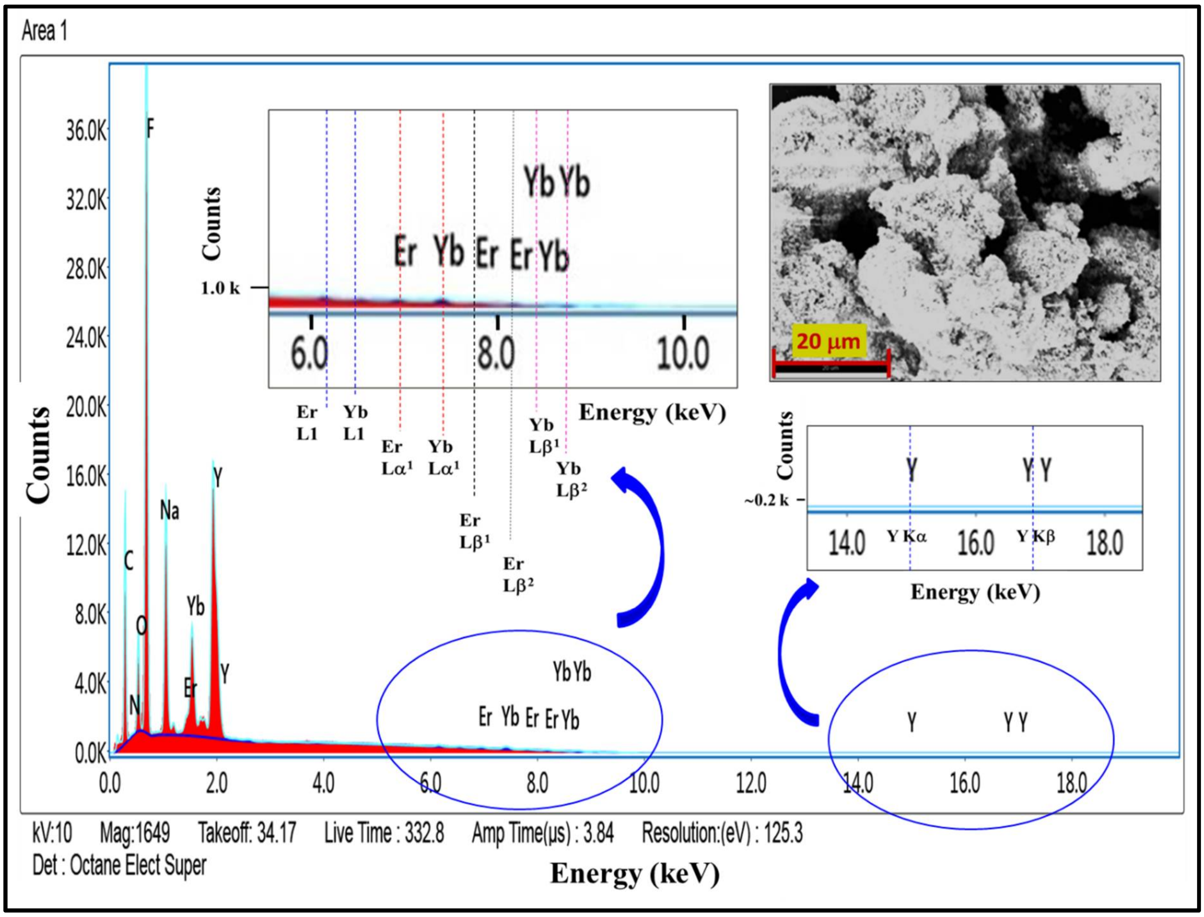

Figure S3. (**a**): EDAX survey spectrum of NaYF4:Yb,Er-PLGA-Nanocurcumin complex shows the presence of Na, Y and F (host ions); Yb and Er (dopant ions), and organic species C, O and N (from PLGA polymer and Nanocurcumin).

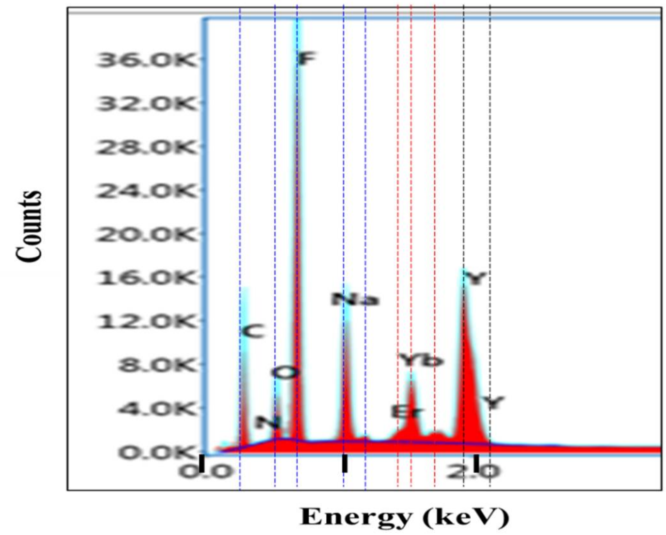

Chemical elements present in NaYF4:YbEr-PLGA-Nanocurcumin (EDAX energy range 0 to 3 keV)			
Element	Weight %	Atomic %	Energy (keV)
C Kα	18.02	37.62	0.277
N Kα			0.067
O Kα	4.05	6.35	0.525
F Kα	26.15	34.52	0.677
Na Kα	10.19	11.12	1.040
Na Kβ			1.067
Er Mα	1.22	0.18	1.405
Yb Mα	9.34	1.35	1.526
Yb Mβ			1.567
Yb Mγ			1.765
Y Lα¹	30.97	8.74	1.922
Y Lβ¹			1.995
Y Lβ²			2.075

Figure S3 (**b**): EDAX spectrum in the range 0 to 3 keV shows strong peaks for the elements F, Y, and Na ion and less intense peaks from 1.4 and 2 kev related to M-shell X-ray emission energy lines for dopant Yb and Er ions. The organic species C, N, O are from PLGA polymer and curcumin drug.

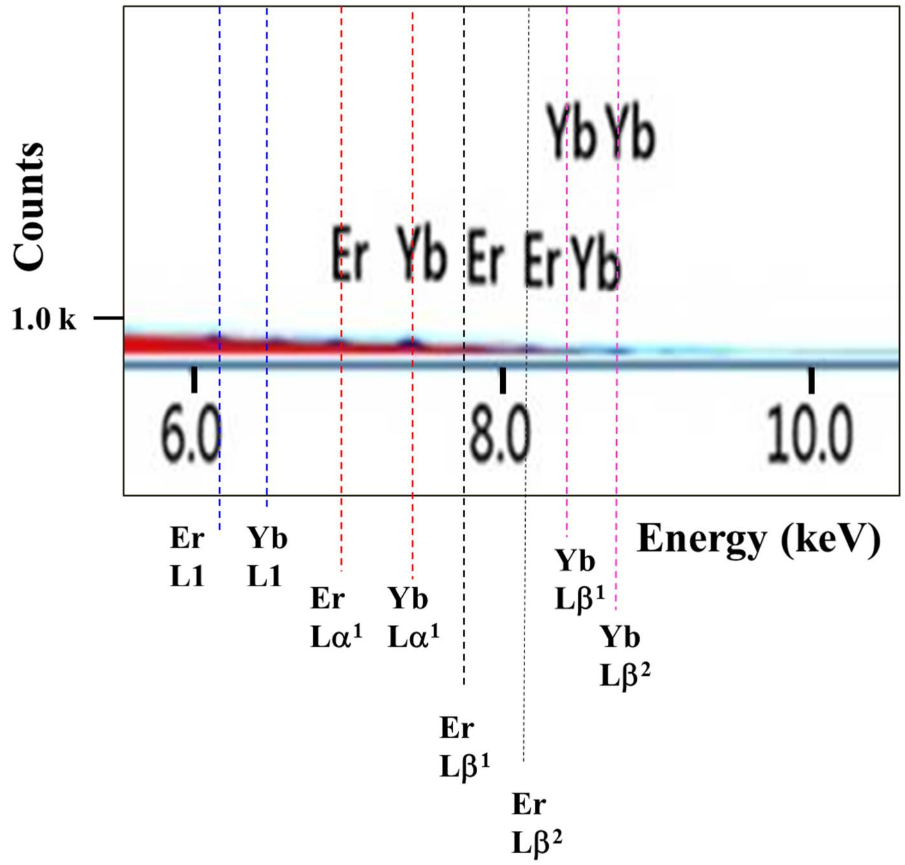

Chemical elements present in NaYF4:YbEr-PLGA-Nanocurcumin (in the EDAX energy range 6 to 10 keV) L-series keV	
Element	Energy (keV)
Er Ll	6.151
Yb Ll	6.543
Er $L\alpha^{1}$	6.946
Yb $L\alpha^{1}$	7.413
Er $L\beta^{1}$	7.808
Er $L\beta^{2}$	8.187
Yb $L\beta^{1}$	8.398
Yb $L\beta^{2}$	8.755

Figure S3 (**c**): EDAX spectrum in the range 6 to 10 keV shows very weak peaks for Yb and Er due to their L-shell X-ray emission energy lines. (EDAX energy positions are assigned by referring AMTEK−EDAX interactive energy table available in the following websites)
https://www.edax.com/resources/interactive-slide-rule-by-atomic-number
https://physics.nist.gov/PhysRefData/XrayTrans/Html/search.html

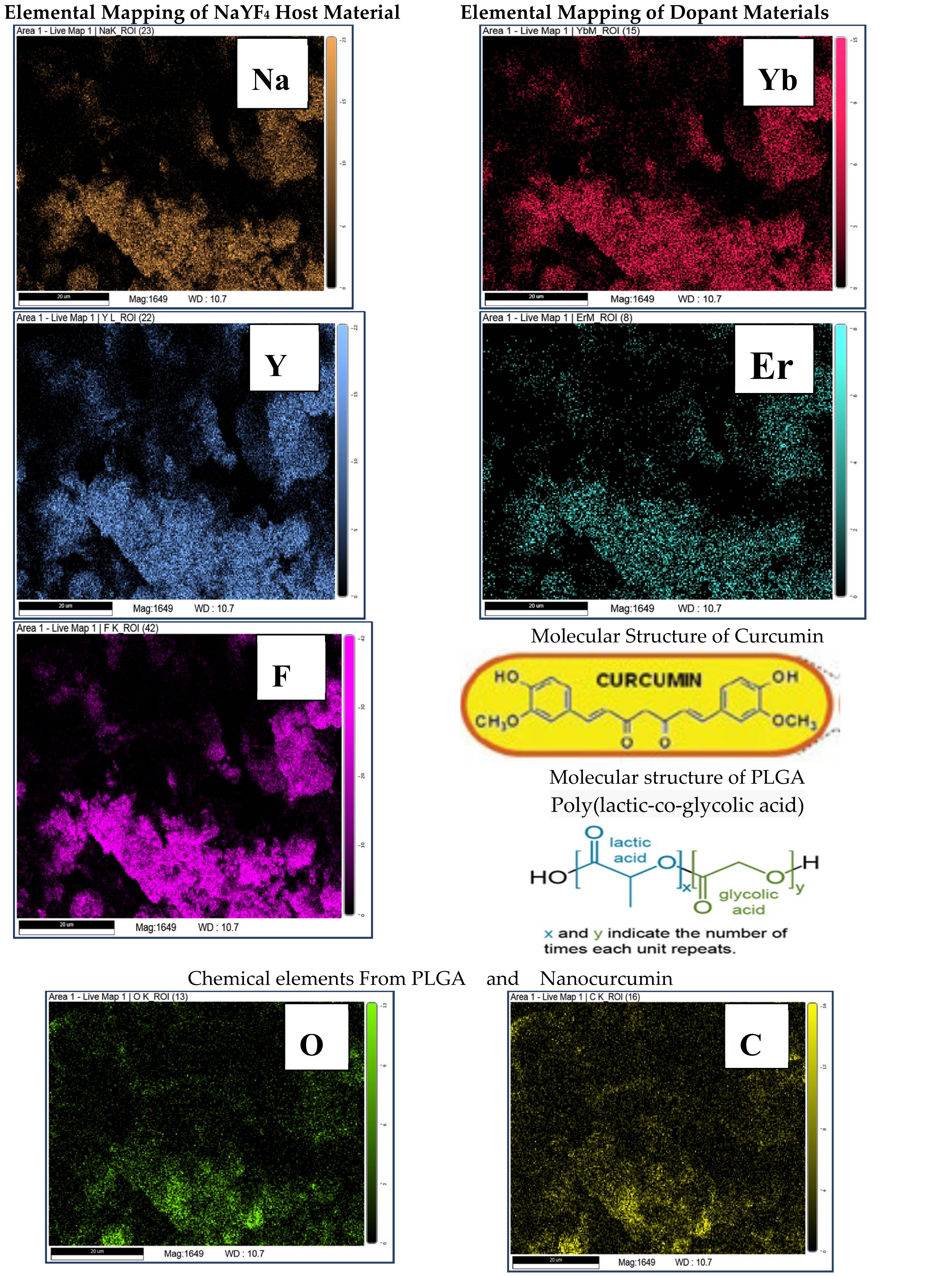

Figure S4. EDAX elemental mapping of Na, Y, F, C, O, Er, Yb elements in the $NaYF_4$:Yb,Er-PLGA-Nanocur complex.

4. Zeta potential analysis

The dynamic light scattering based zeta potential analysis is useful to understand about the surface charge properties of nanoparticles, estimation on the electrostatic potential of colloidal nanoparticle and hence helpful to predict the stability of nanoparticles for bio applications. This colloidal nano suspension has positive or negative electric charge [8,9]. Figure S5 (a–e) shows the zeta potential of the nanocurcumin, UCNP and UCNP-nanocurcumin complexes in the neutral pH water solution. The nanocurcumin displayed the highest negative zeta potential of -60.3 mV that imply high stability; such property is beneficial to conjugate the drug molecule with UCNPs for increased stability in the biocelluar system. The curcumin drug free UCNP1 ($NaYF_4$:Yb,Er) and UCNP2 ($NaYF_4$:Yb,Tm) shows a reduction in zeta potential of −28.3 mV and −39.5 mV respectively. It reveals that UCNPs with moderate zeta potential indicates sufficient stability to use it as drug nanocarrier [10]. Surprisingly, Zeta potential gets increased when UCNPs is functionalized with PLGA. The UCNP1–PLGA–nanocur and UCNP2–PLGA–nanocur exhibits a high negative zeta potential of −42.9 mV and −59 mV respectively. This improved zeta potential indicates good colloidal stability. It specifies successful conjugation between the nanocurcumin drug and UCNPs due to the effective functionalization through carboxylic functional groups of PLGA [11]. Considering the fact that maximum cell membranes hold negative charge, the present zeta potential of nanocurcumin drug loaded UCNP-PLGA complexes are able to improve biodistribution. Therefore, nanocurcumin incorporated UCNP-PLGA nanocomplex which displays a negative zeta potential could give excellent chemical stability for drug delivery and various biological applications [12, 9]. The zeta potential values are given in Table S1.

Table S1. Zeta potential values of UCNP-Nanocurcumin complexes.

S.No.	Materials name	Zeta potential
1.	Nanocurcumin	−60.3 mV
2.	UCNP1 ($NaYF_4$:Yb,Er)	−28.3 mV
3.	UCNP1-PLGA-nanocur	−42.9 mV
4.	UCNP2 ($NaYF_4$:Yb,Tm)	−39.5 mV
5.	UCNP2-PLGA-nanocur	−59.0 mV

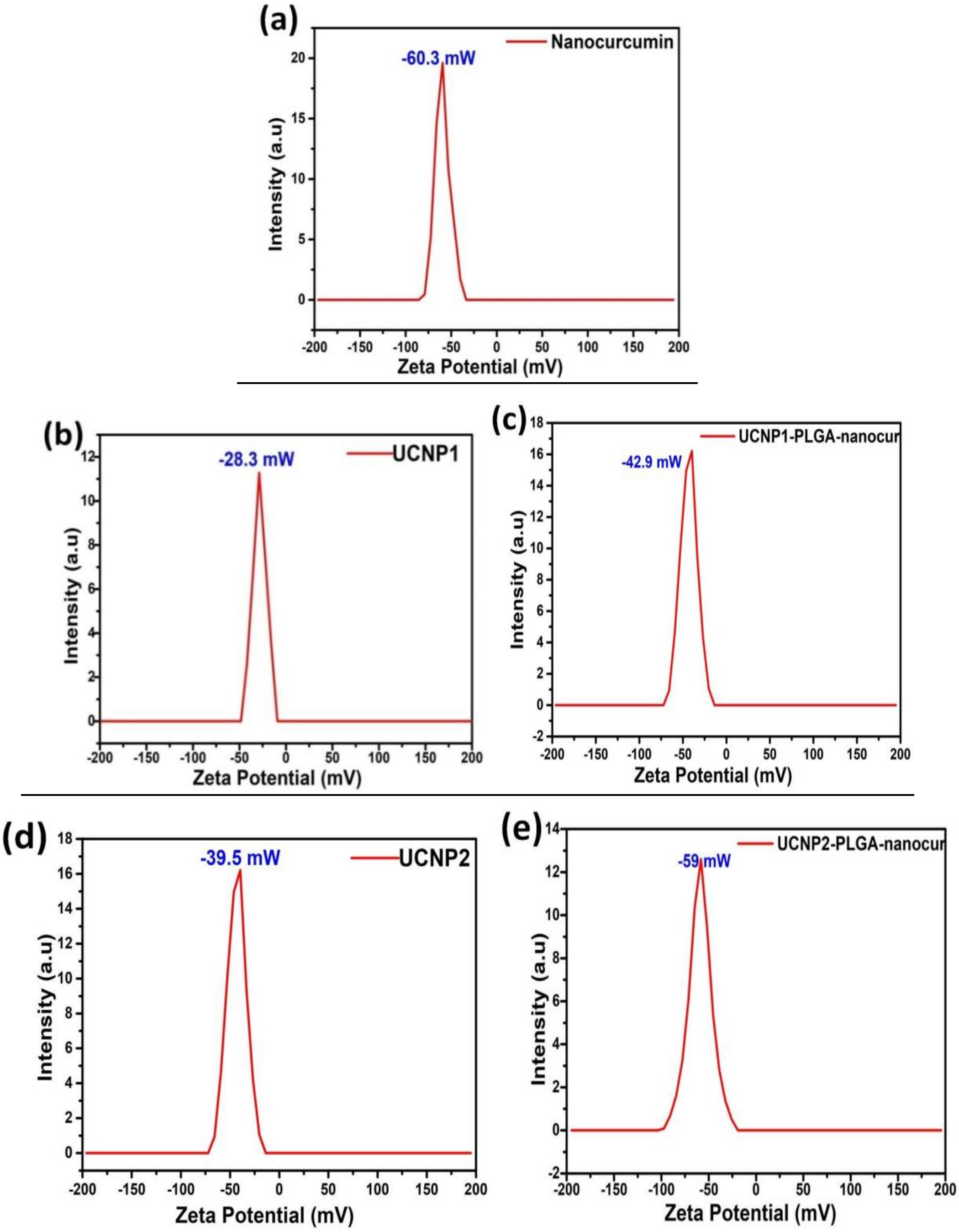

Figure S5. Zeta potential profiles of the nanocurcumin, UCNP1 ($NaYF_4$:Yb,Er), UCNP1-PLGA-nanocurcumin, UCNP2 ($NaYF_4$:Yb,Tm) and UCNP2-PLGA-nanocurcumin complex (taken in aqueous solution at pH 7).

4. References

1. D. Carvalho, K. Takeuchi, R. Geraldine, C. Moura, M. Torres, Production, solubility and antioxidant activity of curcumin nanosuspension, Food Science and Technology (Campinas), 35 (2015) 115−119.

2. R.S. Pandit, S.C. Gaikwad, G.A. Agarkar, A.K. Gade, M. Rai, Curcumin nanoparticles: physico-chemical fabrication and its in vitro efficacy against human pathogens, 3 Biotech, 5 (2015) 991−997.

3. M. Ghosh, A. Singh, W. Xu, T. Sulchek, L. Gordon, R. Ryan, Curcumin nanodisks: Formulation and characterization, Nanomedicine: nanotechnology, biology, and medicine, 7 (2011) 162−167.

4. M. Abirami, M. Raja, P. Mekala, P. Visha, Preparation And Characterisation of Nanocurcumin Suspension International Journal of Science and Technology, Vol. 7, (2018) 100−103.

5. Rajasekar, Facile synthesis of curcumin nanocrystals and validation of its antioxidant activity against circulatory toxicity in wistar rats, Journal of nanoscience and nanotechnology, 15 (2015) 4119−4125.

6. Y. Ye, Y. Li, F. Fang, Upconversion nanoparticles conjugated with curcumin as a photosensitizer to inhibit methicillin-resistant Staphylococcus aureus in lung under near infrared light, International journal of nanomedicine, 9 (2014) 5157−5165.

7. Z. Sayyar, H.J. Malmiri, Preparation, Characterization and Evaluation of Curcumin Nanodispersions Using Three Different Methods – Novel Subcritical Water Conditions, Spontaneous Emulsification and Solvent Displacement, Zeitschrift für Physikalische Chemie, 233 (2019) 1485−1502.

8. R.S. Pandit, S.C. Gaikwad, G.A. Agarkar, A.K. Gade, M. Rai, Curcumin nanoparticles: physico-chemical fabrication and its in vitro efficacy against human pathogens, 3 Biotech, 5 (2015) 991−997.

9. R. Singh, P. Kesharwani, N.K. Mehra, S. Singh, S. Banerjee, N.K. Jain, Development and characterization of folate anchored Saquinavir entrapped PLGA nanoparticles for anti-tumor activity, Drug development and industrial pharmacy, 41 (2015) 1888−1901.

10. R. Singh, J.W. Lillard Jr, Nanoparticle-based targeted drug delivery, Experimental and molecular pathology, 86 (2009) 215−223.

11. G.D. Paka, C. Ramassamy, Optimization of curcumin-loaded PEG-PLGA nanoparticles by GSH functionalization: investigation of the internalization pathway in neuronal cells, Molecular pharmaceutics, 14 (2016) 93–106.

12. Y. Ye, Y. Li, F. Fang, Upconversion nanoparticles conjugated with curcumin as a photosensitizer to inhibit methicillin-resistant Staphylococcus aureus in lung under near infrared light, International journal of nanomedicine, 9 (2014) 5157–5165.

Courtesy: Nanocurcumin Structure in Figure S4

V. Rodov1, I. Shlar1,2, B. Horev1, Y. Vinokur1, M. Shemesh1, E. Poverenov1, S. Droby1, A. Buslovich1,4, A. Friedlander1,2, R. Choudhary3, P. Kohli3, J.D. Haddock3, G. Amirian4, I. Perelshtein4, A. Gedanken4, NANOCURCUMIN for food safety and quality, VOLCANI Voice , Vol.4, Sept.2017, Page 26–29

Courtesy: PLGA Structure in Figure S4

https://akinainc.com/polyscitech/PLGA/PLGA.php

Jin-Seok Choi, Kyuhwa Seo, Jin-Wook Yoo, Recent advances in PLGA particulate systems for drug delivery, Journal of Pharmaceutical Investigation (2012) 42:155–163 DOI 10.1007/s40005-012-0024-5

Funding: All the authors thank the Department of Science and Technology, India (Ref.:INT/RUS/RFBR/P-333 Dated 02.01.2019) and Russian Foundation for Basic Research (No.18-54-45034 IND), Russia for the research grant support.
